# The Influence of Drinking Water Intake on Pollutant Levels in the Human Body: Evidence from NHANES Data

**DOI:** 10.3390/life15060956

**Published:** 2025-06-13

**Authors:** Chenxu Dai, Ziyi Qian, Linjie Yang, Siyan Chen, Hongfei Hu, Xia Huo

**Affiliations:** 1 Laboratory of Environmental Medicine and Developmental Toxicology, Guangdong Key Laboratory of Environmental Pollution and Health, College of Environment and Climate, Jinan University, Guangzhou 511443, China; dcxdyx@stu2022.jnu.edu.cn (C.D.);; 2 Department of Public Health and Preventive Medicine, School of Medicine, Jinan University, Guangzhou 510632, China

**Keywords:** water intake, pollutant, health

## Abstract

Most studies have focused on the levels of pollutants in drinking water and the health risks they pose. However, no studies have reported the effects of drinking water intake on pollutant levels in the human body. Therefore, this study collected data from National Health and Nutrition Examination Survey database to provide statistical evidence for the relationship between water intake and human pollutant levels. We analyzed 95 pollutants in human urine, blood, and serum. The study found that 82% (65/79) of urine pollutants unadjusted for creatinine showed a stable negative correlation with water intake, primarily due to the urine dilution effect caused by increased water consumption. Water intake was negatively correlated with cadmium, m-/p-xylene, and toluene in blood, but positively correlated with blood total mercury and methyl mercury. In summary, the habit of drinking more water may be beneficial to reduce levels of most pollutants in human urine (unadjusted for creatinine) and a small part in blood. Only a few pollutants, including total mercury and methyl mercury in blood, as well as benzophenone-3 in urine (both creatinine-adjusted and unadjusted), are positively related to water intake. The underlying mechanisms by which water intake influences pollutant levels in the human body require further investigation.

## 1. Introduction

Drinking plenty of water is generally considered a healthy habit. It was once popular to drink at least eight 8 oz glasses of water a day (1893 mL per day), although there is no scientific research to support it [1]. Water is an essential and major component of the human body. It is involved in most physiological processes and is vital to human life activities [2,3]. Limited evidence shows that drinking more water has benefits for the human body, such as improved cognitive ability, weight loss, reduced kidney stone events, prevention of migraines and urinary tract infections, diabetes control, improved low blood pressure, and reduced risk of cancer and heart disease [1,4,5,6,7].

Environmental pollutants enter the human body through various exposure pathways, including dietary intake, breathing, and skin contact [8]. Environmental pollutants are commonly found in the human body, posing health threats to the human body [8]. Reducing the levels of pollutants in the human body is undoubtedly a concern. There are currently various pollutants in drinking water [9,10]. If consumed in large amounts over many years, the levels of pollutants in the human body may increase [1]. However, increased drinking water intake may also promote the excretion of toxic substances in the human body [11]. Some studies also suggest that increased water intake dilutes blood and urine [12,13], potentially affecting pollutant levels in these fluids. Therefore, it is necessary to explore whether the habit of drinking more water has public health significance in reducing the levels of pollutants in the human body.

To investigate this, we analyzed data on human pollutant levels and drinking water intake from National Health and Nutrition Examination Survey (NHANES) to explore the association between drinking water intake and human pollutant levels using population-based analyses. The analysis included the following pollutants: metals, per- and polyfluoroalkyl substances, volatile organic compounds and their metabolites, polycyclic aromatic hydrocarbon metabolites, phthalates and plasticizer metabolites, personal care and consumer product chemicals and metabolites, pesticide and metabolites, perchlorate, nitrate, and thiocyanate.

## 2. Methods

### 2.1. Study Population

NHANES is a survey on the health and nutritional status of the American population that has been conducted every two years in recent years, including questionnaires, physical examinations, laboratory tests, dietary interviews, etc. For more information about NHANES and the data collected over the years, please visit the official website (https://www.cdc.gov/nchs/nhanes/about/index.html, accessed on 15 February 2025, and https://wwwn.cdc.gov/nchs/nhanes/default.aspx, accessed on 15 February 2025). Participants underwent a physical examination, provided biological samples (blood and urine), and completed a 24 h dietary recall interview at the Mobile Examination Center (MEC). The dietary interview also recorded the amount of water consumed the previous day, including plain tap water, water from drinking fountains, water coolers, bottled water, and spring water. For this analysis, we included only participants who reported that their food and drink yesterday was consistent with their usual habits, ensuring that their recorded water consumption more accurately reflected their daily intake rather than short-term variations. Since water intake is influenced by age and body weight, we focused on participants aged 20 to 50 years, whose water consumption tends to be relatively stable. Participants from the years 2011 to 2018 were included in the study. For each pollutant, the number of participants screened and the corresponding years are detailed in Table S1.

### 2.2. Quantification of Water Intake

According to the NHANES website, “A set of measuring guides (various glasses, bowls, mugs, bottles, household spoons, measuring cups and spoons, a ruler, thickness sticks, bean bags, and circles) was available in the MEC dietary interview room for the participant to use for reporting amounts of foods” (https://wwwn.cdc.gov/Nchs/Data/Nhanes/Public/2013/DataFiles/DR1TOT_H.htm, accessed on 15 February 2025). More detailed information can be found at https://archive.cdc.gov/www_cdc_gov/nchs/nhanes/measuring_guides_dri/measuringguides.htm (accessed on 15 February 2025).

### 2.3. Detection of Pollutant Levels in the Human Body

Detailed methods for testing 95 pollutants in blood, serum, and urine can be found in the NHANES laboratory method files, such as the 2013–2014 laboratory method files (https://wwwn.cdc.gov/nchs/nhanes/continuousnhanes/labmethods.aspx?BeginYear=2013, accessed on 15 February 2025). Pollutant concentration below the detection limit was represented by the detection limit divided by the square root of 2.

### 2.4. Statistical Analysis Methods

We applied four methods to analyze the data and assess the stability of the results.

Method 1: Drinking water consumption was divided into five levels based on quintiles: The lowest 20% (0–20%) was classified as 1, 20–40% as 2, 40–60% as 3, 60–80% as 4, and the highest 20% (80–100%) as 5. These divided levels (1, 2, 3, 4 and 5) were used in place of water intake. The association between transformed water intake and pollutant levels was analyzed using linear regression models, adjusting for age, sex, and weight. This approach helps mitigate the influence of extreme water intake values on the analysis.

Method 2: Both drinking water consumption and pollutant levels were divided into five levels based on quintiles, following the same classification method as in Method 1. The association between the transformed water intake and transformed pollutant levels was then analyzed using linear regression models adjusted for age, sex, and weight. This method not only accounts for extreme values in water consumption but also addresses the impact of extreme pollutant levels on the analysis.

Method 3: Since body weight may be an important reason for the difference in water intake between individuals, water intake per unit of body weight may better represent the individual’s water intake. Both water intake per unit weight and pollutant levels were divided into five levels based on quintiles, following the same classification method as in Method 1. The association between transformed water intake and transformed pollutant levels was analyzed using linear regression models, adjusting for sex and age.

Method 4: According to the classification of water intake by Method 1, participants with water intake levels 1 and 2 were defined as the low-water intake group, and participants with water intake levels 4 and 5 were defined as the high-water intake group. Pollutant levels between these two groups were compared using the Mann–Whitney U test. Differences were evaluated based on mean rank and *p*-values.

For a given pollutant, if Methods 1, 2, and 3 all indicate a significant positive correlation with water intake, and Method 4 further confirms that the high-water intake group has a significantly higher pollutant level, then the pollutant is considered to be significantly positively correlated with water intake. If three of the methods produce consistent and significant results, the pollutant is also considered to be significantly associated with water intake. However, if only one or two methods yield consistent and significant associations, the correlation between the pollutant and water intake is deemed unstable.

Water intake affects urine volume, which in turn influences urine concentration. Therefore, the analysis of pollutant concentrations in urine needs to account for the effect of urine dilution. Since creatinine is produced in the human body at a relatively stable rate and excreted into urine at an approximately constant rate, its concentration in urine can serve as an indicator of urine dilution. Pollutant concentrations in urine are often adjusted using creatinine concentration, as described below [14,15]:

$$C_{adjusted}=\frac{C_{measured}}{Creatinine\;Concentration\;(mg/dL)}$$

C_measured_ is the original concentration of the pollutant in urine, C_adjusted_ is the creatinine-adjusted concentration.

The association between water intake and urinary pollutant concentrations—both creatinine-adjusted and unadjusted—was analyzed using the four methods mentioned above.

All analyses were performed using SPSS 25. *p* < 0.05 was considered statistically significant.

## 3. Results and Discussion

This study collected 193 pollutants, of which only 95 pollutants with a detection rate of 70% or higher were included in the analysis—79 in urine and 16 in blood and serum (Table S1). The detection rate of volatile organic compounds (VOCs) in blood was generally low, and only 2 of the 51 blood VOCs had a detection rate of more than 70% (Table S1). Water intake and concentrations of pollutants have been summarized in Table S2. The average water intake of all participants yesterday was 1329.78 g.

### 3.1. Human Biomonitoring of Pollutants

Human biomonitoring of pollutants requires the selection of appropriate biomarkers and human samples. Biomarkers integrate multiple exposure sources and can be used to assess the level of pollutant exposure in the human body and help conduct epidemiological studies. Biomarkers can be pollutants themselves, metabolites of pollutants, or products of the interaction between pollutants and biological molecules [16]. Blood and urine are the most commonly used matrices for human biomonitoring. Other human matrices, such as hair, teeth, nails, lung gas, and saliva, have also been used for human biomonitoring. However, these matrices have some disadvantages, such as poor availability, high individual variability, and difficulty in collection and storage, which have prevented them from being widely used [17].

Per- and polyfluoroalkyl substances (PFASs) have a long half-life in the human body, which can reach several years [18,19]. Blood (including whole blood, serum, or plasma) is an excellent matrix for assessing human PFAS exposure [20]. Polycyclic aromatic hydrocarbons (PAHs) are excreted quickly, usually within a few days. The hydroxylated metabolites of PAHs in urine are usually used as biomarkers of internal exposure to assess recent exposure [21,22]. Phthalates also have a short half-life in the human body and are rapidly metabolized into monoester forms and excreted in urine. Therefore, the metabolites of phthalates in urine are often used as biomarkers. The content of phthalate metabolites in urine reflects the short-term exposure of the human body [23,24]. VOCs are metabolized quickly by the human body, with a short half-life of only a few hours. VOCs in the blood can only represent the most recent exposure level. Due to the high volatility of VOCs, they may diffuse into the atmosphere during sample collection and processing. Testing VOCs themselves may underestimate the true exposure level. The more stable VOC metabolites excreted in urine can also be used as biomarkers [25]. For some metals, both blood and urine can be used as test matrices to reflect recent or long-term exposure levels [26]. Urine is one of the excretion pathways of pollutants, and the content of pollutants in urine can also reflect the amount of pollutant excretion. Daily water intake may affect the level of biomarkers reflecting long-term or short-term pollutant exposure.

### 3.2. Pollutants in Drinking Water

PFASs have been found in drinking water throughout the United States [27]. PFASs have been found in both tap water and bottled water, and ingestion of PFASs in drinking water does not pose a health risk [28]. PFASs are present in both raw and treated water samples from public water supplies, and the concentrations of the most common perfluorooctanoic acid (PFOA) and perfluorooctane sulfonate (PFOS) do not exceed the prescribed protection values [29]. PAHs can be detected in tap water in Chinese kitchens, and the carcinogenic risk caused by drinking water intake is within an acceptable range [30]. The presence of PAHs is also found in Turkish drinking water studies, and no concentrations exceeding the standard are found [31]. Phthalates have been found in drinking water in many countries, and no health risks have been found through drinking water intake [32,33,34,35]. Testing of drinking water samples from 2401 household wells in the United States found that VOCs were present in 65% of the samples, and 1.2% of the samples had VOC concentrations exceeding the limit [36]. Pesticide residues have been found in drinking water in many countries around the world, posing a high health risk to the public [37]. Drinking water also contains pollutants such as microplastics [38] and heavy metals [39]. Long-term intake of various pollutants in drinking water may increase the body’s exposure to pollutants.

### 3.3. Urine Pollutant Analysis

Among urine pollutants unadjusted for creatinine, 82% (65/79) were negatively correlated with water intake. However, after creatinine adjustment, only 29% (23/79) remained negatively correlated with water intake (Table 1). This indicates that creatinine plays a role in adjusting for the dilution effect and that urine dilution is the primary reason for the negative correlation observed between unadjusted urine pollutants and water intake. In addition, 42% (33/79) of urine pollutants were significantly negatively correlated with water intake before creatinine adjustment, but were no longer correlated with water intake after creatinine adjustment, further confirming the dilutive effect of water intake on unadjusted urine pollutant levels.

In the creatinine-adjusted analysis, the concentrations of total arsenic, dimethylarsinic acid, inorganic mercury, cesium, molybdenum, thallium, uranium, mono-ethyl phthalate, benzophenone-3, dimethylthiophosphate, 2,4-dicholorphenoxyacetic acid, 3,5,6-trichloropyridinol, perchlorate, nitrate, and N-Acetyl-S-(n-propyl)-L-cysteine in urine were positively correlated with the amount of water consumed (Table 1). Increased water consumption may facilitate the excretion of these substances. However, drinking water can also contain pollutants such as heavy metals [40], meaning that increased water intake may lead to increased pollutant intake. Without comparing excretion with intake, it remains unclear whether increased water intake reduces levels of pollutants in the body. On the other hand, since the levels of pollutants in urine can serve as biomarkers of pollutant exposure levels, the positive correlation between creatinine-adjusted urine pollutants and water intake may also mean that the exposure level of pollutants increases with increasing water intake. Various pollutants in drinking water may be responsible for the increased exposure to pollutants as water consumption increases.

### 3.4. Potential Benefits of Drinking More Water

Increasing water intake can significantly reduce the mutagenicity of urine to prokaryotic cells, likely due to the dilution of urine and its carcinogenic compounds, thereby minimizing their contact with cells [41]. Less fluid intake, higher urine concentration, and less frequent urination are potential risk factors for bladder cancer [42]. A study in men found that increased fluid intake is associated with a reduced risk of bladder cancer [43]. This may be because increased water intake reduces urine concentration, increases urination frequency, and reduces the contact between carcinogenic or toxic substances in urine and the bladder epithelium [42,43]. The urinary system is directly exposed to pollutants during the production, storage, and excretion of urine, which can easily affect the urinary organs including the kidneys and bladder [44]. Pollutants such as heavy metals, aromatic amines, and polycyclic aromatic hydrocarbons are risk factors for bladder cancer [45,46]. Studies have shown that reducing heavy metal exposure can prevent urinary system-related diseases and reduce the incidence of kidney cancer and bladder cancer [44]. Adequate water intake reduces urine concentration, making kidney function more efficient and helping to protect the kidneys [47]. In a conventional low-pollution environment, it may be difficult to further reduce exposure to pollutants such as heavy metals, but reducing the concentration of urinary pollutants by increasing the amount of drinking water may also play a role in preventing urinary system-related diseases and bring potential health benefits.

### 3.5. Blood Pollutant Analysis

Among the 16 pollutants in blood and serum, only 19% (3/16) were negatively correlated with water intake (Table 1), indicating that the dilution effect of drinking water on blood did not seem to be significant.

The detection rates of three forms of mercury, namely inorganic mercury, ethyl mercury, and methyl mercury, were 22.2%, 1.9%, and 79.6%, respectively (Table S1). Methyl mercury accounted for a large proportion of total mercury. Both methyl mercury and total mercury in blood were positively correlated with water intake, and creatinine-adjusted urinary inorganic mercury was also positively correlated with water intake (Table 1). This implies that human mercury exposure levels may increase with increased water consumption. A study of tap water in households in low-income communities in the United States found that mercury concentrations in 50% of the tested water samples exceed the US EPA drinking water standard [48]. Aquifers are important sources of drinking water in the United States, and mercury is a common contaminant in them [49]. Therefore, the positive correlation between water intake and mercury level in blood and urine may be caused by the bioaccumulation of mercury from drinking water. Future research should pay more attention to mercury pollution in drinking water, and perhaps stricter drinking water quality standards are needed to reduce mercury exposure. A well-known example of mercury poisoning is the 1956 Minamata incident in Japan, where severe methyl mercury poisoning occurred due to the consumption of seafood contaminated by industrial mercury discharge [50]. Mercury is a non-essential and toxic metal, and excessive exposure to mercury will have adverse effects on the human nervous system, cardiovascular system, reproductive system, lungs, and kidneys [51,52].

We found that blood cadmium levels were significantly negatively correlated with water intake (Table 1), but creatinine-adjusted urinary cadmium was not associated with water intake. Cadmium in urine reflects long-term exposure levels, while cadmium in blood reflects short-term exposure [26]. Therefore, water consumption may not affect cadmium levels in the long term, but in the short term, increased water consumption may reduce blood cadmium concentrations, we hypothesize, possibly by diluting the blood.

Two volatile organic compounds in blood—m-/p-xylene and toluene—are negatively associated with water intake (Table 1). Xylene includes three isomers: o-xylene, m-xylene, and p-xylene. Due to the highly similar physicochemical properties of m-xylene and p-xylene, they are often reported together as m-/p-xylene. Both xylene and toluene have shorter biological half-lives and are rapidly metabolized and eliminated from the human body [53,54]. Increased water intake may accelerate their metabolic and excretory process. Similar to m-/p-xylene, its creatinine-adjusted metabolites in urine—3-methylhippuric acid and 4-methylhippuric acid—are also negatively correlated with water intake, which may mean that the body’s m-/p-xylene level decreases with increasing water intake.

Perfluorinated and polyfluoroalkyl substances (PFAS) are highly chemically stable and environmentally persistent, with a long half-life in the human body and slow metabolism and excretion [55]. Although PFAS are present in drinking water and are highly bioaccumulative [56,57], our study did not find a significant association between water intake and serum PFAS concentrations (Table 1). Consistent with the results of a previous review [56], PFAS exposure from drinking water intake does not seem to be a concern. In addition, the high hydrophobicity of PFAS [58] and their strong affinity for proteins [20,57] may explain why the levels of PFAS are not affected by water intake. Compared with VOCs (m-/p-xylene and toluene), which are metabolized and excreted quickly and are inversely related to water consumption, persistent pollutants (PFAS), which are metabolized and excreted slowly and have a long half-life, are not related to water consumption. The characteristics of the pollutants and the mechanisms of metabolism and excretion may account for the different associations between water intake and pollutant levels.

## 4. Strengths and Limitations

The large sample size is an advantage of this study (Table S2). The mechanisms underlying the relationship between water intake and pollutant levels require further investigation. This is a cross-sectional study, so it is difficult to draw a causal relationship. Future studies with improved study designs (e.g., randomized controlled trials) may help clarify the causal relationship between water consumption and pollutant concentrations in the human body. It is also important to note that creatinine excretion can be influenced by factors such as age, sex, and the time of urine collection (morning, afternoon, or evening) [14]. Although creatinine is commonly used to adjust for urine dilution when analyzing urinary pollutant concentrations, this method has certain limitations. Therefore, analyses based on creatinine-adjusted urinary pollutant levels should be interpreted with caution. Interestingly, one study found that drinking cola drinks can reduce blood lead levels in animal models [59]. Future studies can also focus on the impact of cola and other beverages on human pollutant levels.

## 5. Conclusions

By analyzing the association between drinking water intake and human pollutant levels, we found that some pollutants are negatively correlated with drinking water intake, and increased drinking water intake may help reduce the levels of these pollutants. However, we also found that some pollutants are positively correlated with drinking water intake, and increased drinking water intake may lead to increased exposure to these pollutants. Our study provides statistical evidence for the potential benefits of drinking water intake on human pollutant levels and the human exposure risk of trace pollutants in drinking water.

## Figures and Tables

**Table 1:** Summary table of the relationship between drinking water and pollutant levels.

	Pollutants	Unadjusted Creatinine	Adjusted Creatinine
**Metals in blood**			
	Lead	Not	/
	Cadmium	-	/
	Manganese	Not	/
	Total mercury	+	/
	Cobalt	Not	/
	Methyl mercury	+	/
**Per- and polyfluoroalkyl substances in serum**			
	Perfluorodecanoic acid	Not	/
	Perfluorooctanoic acid	Not	/
	Perfluorooctane sulfonic acid	Not	/
	Perfluorohexane sulfonic acid	Not	/
	Perfluorononanoic acid	Not	/
	Linear-perfluorooctanoic acid	Not	/
	Linear-perfluorooctane sulfonic acid	Not	/
	Perfluoromethylheptane sulfonic acid isomers	Not	/
**Volatile** **organic** **compounds in blood**			
	M-/p-Xylene	-	/
	Toluene	-	/
**Metals in urine**			
	Total arsenic	Not	+
	Dimethylarsinic acid	-	+
	Inorganic mercury	-	+
	Barium	-	Not
	Cadmium	-	Not
	Cobalt	-	Not
	Cesium	-	+
	Molybdenum	-	+
	Lead	-	Not
	Antimony	-	Not
	Strontium	-	Not
	Thallium	-	+
	Tin	-	Not
	Tungsten	-	Not
	Uranium	-	+
	Nickel	-	Not
**Polycyclic aromatic hydrocarbon metabolites in urine**			
	1-Hydroxynaphthalene	-	-
	2-Hydroxynaphthalene	-	-
	3-Hydroxyfluorene	-	-
	2-Hydroxyfluorene	-	-
	3-Hydroxyphenanthrene	-	Not
	1-Hydroxyphenanthrene	-	Not
	2-Hydroxyphenanthrene	-	Not
	1-Hydroxypyrene	-	-
	9-Hydroxyfluorene	-	Not
	4-Hydroxyphenanthrene	-	Not
**Phthalates and plasticizer metabolites in urine**			
	Mono(carboxynonyl) phthalate	-	Not
	Mono(carboxyoctyl) phthalate	-	Not
	Mono-2-ethyl-5-carboxypentyl phthalate	-	Not
	Mono-n-butyl phthalate	-	Not
	Mono-(3-carboxypropyl) phthalate	-	Not
	Mono-ethyl phthalate	Not	+
	Mono-(2-ethyl-5-hydroxyhexyl) phthalate	-	Not
	Mono-(2-ethyl-5-oxohexyl) phthalate	-	Not
	Mono-benzyl phthalate	-	-
	Mono-isobutyl phthalate	-	Not
	Mono-2-hydroxy-iso-butyl phthalate	-	Not
	Mono-3-hydroxy-n-butyl phthalate	-	Not
	Mono-2-ethyl-5-carboxypentylterephthalate	Not	Not
	Mono-2-ethyl-5-hydroxyhexylterephthalate	Not	Not
	Mono-oxoisononyl phthalate	Not	Not
**Personal care and consumer product chemicals and metabolites in urine**			
	Benzophenone-3	+	+
	Bisphenol A	-	-
	Triclosan	Not	Not
	Methyl paraben	Not	Not
	Propyl paraben	Not	Not
	2,5-dichlorophenol	-	-
	2,4-dichlorophenol	-	Not
	Bisphenol S	-	Not
**Pesticide and metabolites in** **urine**			
	**DEET and metabolites in** **urine**		
	3-(Ethlycarbamoyl) benzoic acid	Not	Not
	**Glyphosate in urine**		
	Glyphosate	-	Not
	**Organophosphate** **insecticides and** **dialkyl** **phosphate** **metabolites in urine**		
	Dimethylphosphate	Not	Not
	Diethylphosphate	-	Not
	Dimethylthiophosphate	Not	+
	**Pyrethroids,** **herbicides, and** **organophosphorus** **metabolites in urine**		
	2,4-dicholorphenoxyacetic acid	Not	+
	3-phenoxybenzoic acid	-	Not
	Para-Nitrophenol	-	Not
	3,5,6-trichloropyridinol	-	+
**Perchlorate,** **nitrate, and** **thiocyanate in urine**			
	Perchlorate	-	+
	Nitrate	-	+
	Thiocyanate	-	-
**Volatile** **organic** **compound** **metabolites in urine**			
	2-Methylhippuric acid	-	-
	3-Methylhippuric acid and 4-Methylhippuric acid	-	-
	N-Acetyl-S-(2-carbamoylethyl)-L-cysteine	-	-
	N-Acetyl-S-(N-methylcarbamoyl)-L-cysteine	-	-
	2-Aminothiazoline-4-carboxylic acid	-	Not
	N-Acetyl-S-(benzyl)-L-cysteine	-	Not
	N-Acetyl-S-(n-propyl)-L-cysteine	Not	+
	N-Acetyl-S-(2-carboxyethyl)-L-cysteine	-	-
	N-Acetyl-S-(2-cyanoethyl)-L-cysteine	-	-
	N-Acetyl-S-(3,4-dihydroxybutyl)-L-cysteine	-	-
	N-Acetyl-S-(2-hydroxypropyl)-L-cysteine	-	Not
	N-Acetyl-S-(3-hydroxypropyl)-L-cysteine	-	-
	Mandelic acid	-	-
	N-Acetyl-S-(4-hydroxy-2-butenyl)-L-cysteine	-	-
	Phenylglyoxylic acid	-	-
	N-Acetyl-S-(3-hydroxypropyl-1-methyl)-L-cysteine	-	-
	N-Acetyl-S-(2-hydroxy-3-methyl-3-buten-1-yl)-L-cysteine and N-Acetyl-S-(2-hydroxy-2-methyl-3-buten-1-yl)-L-cysteine	-	-
	N-Acetyl-S-(4-hydroxy-2-methyl-2-buten-1-yl)-L-cysteine	-	-

Note: The associations between pollutants and drinking water intake are summarized through the analysis results in Tables S3 and S4. For detailed analysis data, please refer to the Supplementary Materials; - Represents a stable negative correlation between drinking water and the level of a pollutant; + represents a stable positive correlation between drinking water and the level of a pollutant; Not means the result has no stable significance. Blood pollutant concentrations do not need to be adjusted for creatinine, and the corresponding space is replaced by /.

## Data Availability

The original data presented in the study are openly available at https://wwwn.cdc.gov/nchs/nhanes/Default.aspx (accessed on 15 February 2025).

## Supplementary Materials

The following supporting information can be downloaded at: https://www.mdpi.com/article/10.3390/life15060956/s1; Table S1: Detection limits and detection rates of pollutants in different years; Table S2: Water intake and concentrations of pollutants; Table S3: Associations between water intake and creatinine-unadjusted pollutants analyzed by four methods; Table S4: Associations between water intake and creatinine-adjusted pollutants in urine analyzed by four methods.
