# Supplementary material for: The Influence of Drinking Water Intake on Pollutant Levels in the Human Body: Evidence from NHANES Data
Source: Life (Basel). 2025 Jun 13;15(6):956. doi: 10.3390/life15060956 (PMC12194692; doi:10.3390/life15060956)
Supplement: Supplementary file 1 [file life-15-00956-s001.zip › life-3548237-supplementary.pdf]

Supplementary information

Table S1. Detection limits and detection rates of pollutants in different years.

Table S2. Water intake and concentrations of pollutants.

Table S3. Associations between water intake and creatinine-unadjusted pollutants analyzed by four methods.

Table S4. Associations between water intake and creatinine-adjusted pollutants in urine analyzed by four methods.

Table S1. Detection limits and detection rates of pollutants in different years.

Pollutants	Detection frequency	Number of participants	Detection limits of pollutants in different years			
			2011-2012	2013-2014	2015-2016	2017-2018
**Metals in blood**						
Lead	99.5	4984	0.25 µg/dL	0.07 µg/dL	0.07 µg/dL	0.07 µg/dL
Cadmium	86.5	4984	0.16 µg/L	0.1 µg/L	0.1 µg/L	0.1 µg/L
Manganese	100	4983	1.06 µg/L	0.99 µg/L	0.99 µg/L	0.99 µg/L
Total mercury	87.8	4984	0.16 µg/L	0.28 µg/L	0.28 µg/L	0.28 µg/L
Chromium	12.4	1156	/	/	0.41 µg/L	0.41 µg/L
Cobalt	99.4	1158	/	/	0.06 µg/L	0.06 µg/L
Inorganic mercury	22.2	4979	0.27 µg/L	0.27 µg/L	0.27 µg/L	0.21 µg/L
Ethyl mercury	1.9	4978	0.16 µg/L	0.16 µg/L	0.16 µg/L	0.064 µg/L
Methyl mercury	79.6	4979	0.12 µg/L	0.12 µg/L	0.12 µg/L	0.26 µg/L
**Per- and polyfluoroalkyl substances in serum**						
2-(N-Ethyl-perfluorooctane sulfonamido) acetic acid	3.9	567	0.1 ng/mL	/	/	/
Perfluorodecanoic acid	77.5	2179	0.1 ng/mL	0.1 ng/mL	0.1 ng/mL	0.1 ng/mL
Perfluorooctanoic acid	99.6	567	0.1 ng/mL	/	/	/
Perfluorooctane sulfonic acid	99.8	567	0.2 ng/mL	/	/	/
Perfluorohexane sulfonic acid	98.7	2179	0.1 ng/mL	0.1 ng/mL	0.1 ng/mL	0.1 ng/mL
2-(N-Methyl-perfluorooctane sulfonamido) acetic acid	41.5	2179	0.09 ng/mL	0.1 ng/mL	0.1 ng/mL	0.1 ng/mL

Perfluorobutane sulfonic acid	0.4	1126	0.1 ng/mL	0.1 ng/mL	/	/
Perfluoroheptanoic acid	11.4	1126	0.1 ng/mL	0.1 ng/mL	/	/
Perfluorononanoic acid	96.6	2179	0.08 ng/mL	0.1 ng/mL	0.1 ng/mL	0.1 ng/mL
Perfluorooctane sulfonamide	0	567	0.1 ng/mL	/	/	/
Perfluoroundecanoic acid	49.3	2179	0.1 ng/mL	0.1 ng/mL	0.1 ng/mL	0.1 ng/mL
Perfluorododecanoic acid	8.8	1697	0.1 ng/mL	0.1 ng/mL	0.1 ng/mL	/
Linear-perfluorooctanoic acid	99.1	1053	/	/	0.1 ng/mL	0.1 ng/mL
Branch perfluorooctanoic acid isomers	4.7	1053	/	/	0.1 ng/mL	0.1 ng/mL
Linear-perfluorooctane sulfonic acid	99.6	1053	/	/	0.1 ng/mL	0.1 ng/mL
Perfluoromethylheptane sulfonic acid isomers	98.6	1053	/	/	0.1 ng/mL	0.1 ng/mL
**Volatile organic compounds in blood**						
2,5-Dimethylfuran	21.8	3243	0.011 ng/mL	0.011 ng/mL	0.011 ng/mL	0.011 ng/mL
1,1,1,2-Tetrachloroethane	0	3223	0.04 ng/mL	0.04 ng/mL	0.04 ng/mL	0.04 ng/mL
Hexane	0.8	2980	0.122 ng/mL	0.122 ng/mL	0.122 ng/mL	0.122 ng/mL
1,1-Dichloroethane	0	804	0.01 ng/mL	/	/	/
1,2-Dichlorobenzene	0	3157	0.025 ng/mL	0.025 ng/mL	0.025 ng/mL	0.025 ng/mL
1,1-Dichloroethene	0	827	0.009 ng/mL	/	/	/
1,2-Dichloroethane	2	3235	0.01 ng/mL	0.01 ng/mL	0.01 ng/mL	0.01 ng/mL
cis-1,2-Dichloroethene	0	815	0.01 ng/mL	/	/	/
1,1,2-Trichloroethene	0	822	0.01 ng/mL	/	/	/
trans-1,2-Dichloroethene	0.4	815	0.01 ng/mL	/	/	/
1,3-Dichlorobenzene	0.3	3198	0.025 ng/mL	0.025 ng/mL	0.025 ng/mL	0.025 ng/mL
Tetrachloroethene	8.5	3064	0.048 ng/mL	0.048 ng/mL	0.048 ng/mL	0.048 ng/mL
1,1,2,2-Tetrachloroethane	0.4	791	0.01 ng/mL	/	/	/
Benzene	35.4	3152	0.024 ng/mL	0.024 ng/mL	0.024 ng/mL	0.024 ng/mL

Chlorobenzene	0.2	3236	0.011 ng/mL	0.011 ng/mL	0.011 ng/mL	0.011 ng/mL
Carbon Tetrachloride	0.5	3193	0.005 ng/mL	0.005 ng/mL	0.005 ng/mL	0.005 ng/mL
1,4-Dichlorobenzene	47.3	3191	0.04 ng/mL	0.04 ng/mL	0.04 ng/mL	0.04 ng/mL
1,2-Dibromoethane	0	3210	0.015 ng/mL	0.015 ng/mL	0.015 ng/mL	0.015 ng/mL
Dibromomethane	0.2	815	0.03 ng/mL	/	/	/
1,2-Dichloropropane	0	806	0.01 ng/mL	/	/	/
1,4-Dioxane	0	2476	0.5 ng/mL	0.5 ng/mL	0.5 ng/mL	/
Ethylbenzene	31.4	3166	0.024 ng/mL	0.024 ng/mL	0.024 ng/mL	0.024 ng/mL
Furan	17.8	3175	0.025 ng/mL	0.025 ng/mL	0.025 ng/mL	0.025 ng/mL
Hexachloroethane	0	823	0.011 ng/mL	/	/	/
Isopropylbenzene	0.2	3210	0.04 ng/mL	0.04 ng/mL	0.04 ng/mL	0.04 ng/mL
Methylene Chloride	0.1	3122	0.25 ng/mL	0.25 ng/mL	0.25 ng/mL	0.25 ng/mL
Nitrobenzene	0	3191	0.32 ng/mL	0.32 ng/mL	0.32 ng/mL	0.32 ng/mL
O-Xylene	30.5	3206	0.024 ng/mL	0.024 ng/mL	0.024 ng/mL	0.024 ng/mL
Trichloroethene	0.7	3186	0.012 ng/mL	0.012 ng/mL	0.012 ng/mL	0.012 ng/mL
1,1,1-Trichloroethane	0.2	3180	0.01 ng/mL	0.01 ng/mL	0.01 ng/mL	0.01 ng/mL
1,2,3-Trichloropropane	0	3214	0.04 ng/mL	0.04 ng/mL	0.04 ng/mL	0.04 ng/mL
M-/p-Xylene	70.8	3135	0.034 ng/mL	0.034 ng/mL	0.034 ng/mL	0.034 ng/mL
Heptane	1.3	2350	/	0.1 ng/mL	0.1 ng/mL	0.1 ng/mL
Octane	1.3	2375	/	0.1 ng/mL	0.1 ng/mL	0.1 ng/mL
Bromoform	8	2386	1 pg/mL	0.008 pg/mL	0.008 pg/mL	0.008 pg/mL
Bromodichloromethane	17.4	2395	0.62 pg/mL	0.006 pg/mL	0.006 pg/mL	0.006 pg/mL
Cyclohexane	1.4	2312	/	0.02 ng/mL	0.02 ng/mL	0.02 ng/mL
Chloroform	53.1	2371	2.1 pg/mL	0.008 pg/mL	0.008 pg/mL	0.008 pg/mL
Dibromochloromethane	13.9	2412	0.62 pg/mL	0.005 pg/mL	0.005 pg/mL	0.005 pg/mL
Diethyl Ether	0	2353	/	0.04 ng/mL	0.04 ng/mL	0.04 ng/mL

Ethyl Acetate	1.9	2363	/	0.158 ng/mL	0.158 ng/mL	0.158 ng/mL
Chloroethane	0.3	2385	/	0.045 ng/mL	0.045 ng/mL	0.045 ng/mL
Methyl-tert-butyl ether (MTBE)	1.9	2288	1.4 pg/mL	0.01 pg/mL	0.01 pg/mL	0.01 pg/mL
Methylcyclopentane	1.7	2327	/	0.02 ng/mL	0.02 ng/mL	0.02 ng/mL
aaa-Trifluorotoluene	0	2412	/	0.04 ng/mL	0.04 ng/mL	0.04 ng/mL
Tetrahydrofuran	0.6	2346	/	0.125 ng/mL	0.125 ng/mL	0.125 ng/mL
Toluene	96.2	1676	/	0.025 ng/mL	0.025 ng/mL	/
Vinyl Bromide	0	2403	/	0.045 ng/mL	0.045 ng/mL	0.045 ng/mL
Benzonitrile	7.9	1537	/	/	0.15 ng/mL	0.15 ng/mL
Isobutyronitrile	9.2	1528	/	/	0.04 ng/mL	0.04 ng/mL
Methyl Isobutyl Ketone	0.1	700	/	/	/	0.1 ng/mL
**Metals in urine**						
Total arsenic	98.8	2336	1.25 µg/L	0.26 µg/L	0.26 µg/L	0.23 µg/L
Arsenous acid	51.8	2340	0.48 µg/L	0.12 µg/L	0.12 µg/L	0.12 µg/L
Arsenic acid	3.8	2340	0.87 µg/L	0.79 µg/L	0.79 µg/L	0.79 µg/L
Arsenobetaine	43.9	2340	1.19 µg/L	1.16 µg/L	1.16 µg/L	1.16 µg/L
Arsenocholine	11.2	2340	0.28 µg/L	0.11 µg/L	0.11 µg/L	0.11 µg/L
Dimethylarsinic acid	74	2340	1.8 µg/L	1.91 µg/L	1.91 µg/L	1.91 µg/L
Monomethylarsonic acid	50.8	2340	0.89 µg/L	0.2 µg/L	0.2 µg/L	0.2 µg/L
Trimethylarsine Oxide	2.4	622	0.25 µg/L	/	/	/
Inorganic mercury	65.8	2338	0.05 ng/mL	0.13 ng/mL	0.13 µg/L	0.13 µg/L
Barium	99.5	2338	0.1 µg/L	0.06 µg/L	0.06 µg/L	0.06 µg/L
Cadmium	88.2	2338	0.056 µg/L	0.036 µg/L	0.036 µg/L	0.036 µg/L
Cobalt	99.7	2338	0.048 µg/L	0.023 µg/L	0.023 µg/L	0.023 µg/L
Cesium	100	2338	0.12 µg/L	/	0.086 µg/L	0.086 µg/L
Manganese	39	2338	0.08 µg/L	0.13 µg/L	0.13 µg/L	0.13 µg/L

Molybdenum	100	2337	0.99 µg/L	0.8 µg/L	0.8 µg/L	0.8 µg/L
Lead	97.6	2338	0.08 µg/L	0.03 µg/L	0.03 µg/L	0.03 µg/L
Antimony	71.8	2338	0.041 µg/L	0.022 µg/L	0.022 µg/L	0.022 µg/L
Strontium	100	1836	2.5 µg/L	2.34 µg/L	2.34 µg/L	/
Thallium	99.4	2338	0.02 µg/L	0.018 µg/L	0.018 µg/L	0.018 µg/L
Tin	85.8	2337	0.22 µg/L	0.09 µg/L	0.09 µg/L	0.09 µg/L
Tungsten	83.3	2333	0.026 µg/L	0.018 µg/L	0.018 µg/L	0.018 µg/L
Uranium	76.1	1836	0.0033 µg/L	0.002 µg/L	0.002 µg/L	/
Nickel	90.6	501	/	/	/	0.31 µg/L
**Polycyclic aromatic hydrocarbon metabolites in urine**						
1-Hydroxynaphthalene	99.9	1803	44 ng/L	60 ng/L	60 ng/L	/
2-Hydroxynaphthalene	100	1817	42 ng/L	90 ng/L	90 ng/L	/
3-Hydroxyfluorene	98.4	1828	10 ng/L	8 ng/L	8 ng/L	/
2-Hydroxyfluorene	100	1830	10 ng/L	8 ng/L	8 ng/L	/
3-Hydroxyphenanthrene	97.4	620	10 ng/L	/	/	/
1-Hydroxyphenanthrene	99.7	1830	10 ng/L	9 ng/L	9 ng/L	/
2-Hydroxyphenanthrene	97.7	620	10 ng/L	/	/	/
1-Hydroxypyrene	83.6	1827	10 ng/L	70 ng/L	70 ng/L	/
9-Hydroxyfluorene	100	620	10 ng/L	/	/	/
4-Hydroxyphenanthrene	78	619	10 ng/L	/	/	/
**Phthalates and plasticizer metabolites in urine**						
Mono(carboxynonyl) phthalate	97.3	2306	0.2 ng/mL	0.2 ng/mL	0.2 ng/mL	0.2 ng/mL
Mono(carboxyoctyl) phthalate	99.7	2306	0.2 ng/mL	0.3 ng/mL	0.3 ng/mL	0.3 ng/mL
Mono-2-ethyl-5-carboxypentyl phthalate	99.7	2306	0.2 ng/mL	0.4 ng/mL	0.4 ng/mL	0.4 ng/mL

Mono-n-butyl phthalate	97.1	2306	0.4 ng/mL	0.4 ng/mL	0.4 ng/mL	0.4 ng/mL
Mono-(3-carboxypropyl) phthalate	86	2306	0.2 ng/mL	0.4 ng/mL	0.4 ng/mL	0.4 ng/mL
Mono-ethyl phthalate	99.7	2306	0.6 ng/mL	1.2 ng/mL	1.2 ng/mL	1.2 ng/mL
Mono-(2-ethyl-5-hydroxyhexyl) phthalate	99.3	2306	0.2 ng/mL	0.4 ng/mL	0.4 ng/mL	0.4 ng/mL
Mono-(2-ethyl)-hexyl phthalate	66.1	2306	0.5 ng/mL	0.8 ng/mL	0.8 ng/mL	0.8 ng/mL
Mono-n-methyl phthalate	63.4	621	0.5 ng/mL	/	/	/
Mono-isononyl phthalate	39.2	2306	0.5 ng/mL	0.9 ng/mL	0.9 ng/mL	0.9 ng/mL
Mono-(2-ethyl-5-oxohexyl) phthalate	99	2306	/	0.4 ng/mL	0.4 ng/mL	0.4 ng/mL
Mono-benzyl phthalate	96.8	2306	0.3 ng/mL	0.3 ng/mL	0.3 ng/mL	0.3 ng/mL
Mono-isobutyl phthalate	98.2	2306	0.2 ng/mL	0.8 ng/mL	0.8 ng/mL	0.8 ng/mL
Cyclohexane-1,2-dicarboxylic acid monohydroxy isononyl ester	43.5	2306	/	0.4 ng/mL	0.4 ng/mL	0.4 ng/mL
Mono-2-hydroxy-iso-butyl phthalate	94.9	1107	/	/	0.4 ng/mL	0.4 ng/mL
Cyclohexane-1,2-dicarboxylic acid-mono(carboxyoctyl) ester phthalate	48.5	1107	/	/	0.5 ng/mL	0.5 ng/mL
Mono-3-hydroxy-n-butyl phthalate	70.1	1107	/	/	0.4 ng/mL	0.4 ng/mL
Mono-2-ethyl-5-carboxypentylterephthalate	99.8	513	/	/	/	0.2 ng/mL
Mono-2-ethyl-5-hydroxyhexylterephthalate	95.5	513	/	/	/	0.4 ng/mL
Mono-oxoisononyl phthalate	84	513	/	/	/	0.4 ng/mL
**Personal care and consumer product chemicals and metabolites in urine**						
Benzophenone-3	97.4	1794	0.4 µg/L	0.4 µg/L	0.4 ng/mL	/
Bisphenol A	93	1794	0.4 µg/L	0.2 µg/L	0.2 ng/mL	/
Triclosan	73.6	1794	2.3 µg/L	1.7 µg/L	1.7 ng/mL	/

Butyl paraben	30.4	1794	0.2 µg/L	0.1 µg/L	0.1 ng/mL	/
Ethyl paraben	51.2	1794	1 µg/L	1 µg/L	1 ng/mL	/
Methyl paraben	98.9	1794	1 µg/L	1 µg/L	1 ng/mL	/
Propyl paraben	97.4	1794	0.2 µg/L	0.1 µg/L	0.1 ng/mL	/
2,5-dichlorophenol	97.4	1794	0.2 µg/L	0.1 µg/L	0.1 ng/mL	/
2,4-dichlorophenol	90	1794	0.2 µg/L	0.1 µg/L	0.1 ng/mL	/
Bisphenol F	56.1	1173	/	0.2 µg/L	0.2 ng/mL	/
Bisphenol S	90.7	1173	/	0.1 µg/L	0.1 ng/mL	/
Triclocarban	35	1173	/	0.1 µg/L	0.1 ng/mL	/
**DEET and metabolites in Urine**						
N, N-Diethy-meta-toluamide (DEET)	5.7	1183	0.083 µg/L	0.083 µg/L	/	/
3-(Ethlycarbamoyl) benzoic acid	82.1	1183	0.475 µg/L	0.475 µg/L	/	/
N, N-Diethyl-3-(hydroxymethyl) benzamide	8.8	1178	0.089 µg/L	0.089 µg/L	/	/
**Glyphosate in urine**						
Glyphosate	72.7	1479	/	0.2 ng/mL	0.2 ng/mL	0.1 ng/mL
**Organophosphate insecticides and dialkyl phosphate metabolites in urine**						
Dimethylphosphate	97.2	1664	0.1 ng/mL	/	0.1 ng/mL	0.1 ng/mL
Diethylphosphate	99.1	1677	0.1 ng/mL	/	0.1 ng/mL	0.1 ng/mL
Dimethylthiophosphate	91.3	1672	0.1 ng/mL	/	0.1 ng/mL	0.1 ng/mL
Diethylthiophosphate	64.3	1671	0.1 ng/mL	/	0.1 ng/mL	0.1 ng/mL
Dimethyldithiophosphate	48.8	1677	0.1 ng/mL	/	0.1 ng/mL	0.1 ng/mL
Diethyldithiophosphate	6	1675	0.1 ng/mL	/	0.1 ng/mL	0.1 ng/mL
**Pyrethroids, herbicides, and organophosphorus metabolites in urine**						
2,4-dicholorphenoxyacetic acid	73.9	1773	0.15 µg/L	0.15 µg/L	0.15 µg/L	/

4-fluoro-3-phenoxy-benzoic acid	13.2	1789	0.1 µg/L	0.1 µg/L	0.1 µg/L	/
3-phenoxybenzoic acid	92.5	1770	0.1 µg/L	0.1 µg/L	0.1 µg/L	/
2-isopropyl-4-methyl-pyrimidinol	20.8	1782	0.1 µg/L	0.1 µg/L	0.1 µg/L	/
Para-Nitrophenol	97.1	1750	0.1 µg/L	0.1 µg/L	0.1 µg/L	/
Trans-3-(2,2-dichlorovinyl)-2,2-dimethylcyclopropane carboxylic acid	23.8	1750	0.6 µg/L	0.6 µg/L	0.6 µg/L	/
3,5,6-trichloropyridinol	98.3	1209	/	0.1 µg/L	0.1 µg/L	/
Malathion diacid	15.4	598	/	/	0.5 µg/L	/
**Perchlorate, nitrate and thiocyanate in urine**						
Perchlorate	100	2320	0.05 ng/mL	0.05 ng/mL	0.05 ng/mL	0.05 ng/mL
Nitrate	100	2321	700 ng/mL	700 ng/mL	700 ng/mL	700 ng/mL
Thiocyanate	100	2317	20 ng/mL	20 ng/mL	20 ng/mL	20 ng/mL
**Volatile organic compound metabolites in urine**						
N-Acetyl-S-(1,2-dichlorovinyl)-L-cysteine	0.1	1765	12.6 µg/L	12.6 µg/L	12.6 ng/mL	/
N-Acetyl-S-(2,2-dichlorovinyl)-L-cysteine	0.1	1749	4.7 µg/L	4.7 µg/L	13.9 ng/mL	/
2-Methylhippuric acid	92.8	2236	5 µg/L	5 µg/L	5 ng/mL	5 ng/mL
3-Methylhippuric acid & 4-Methylhippuric acid	99.2	2313	8 µg/L	8 µg/L	8 ng/mL	8 ng/mL
N-Acetyl-S-(2-carbamoylethyl)-L-cysteine	99.9	2276	2.2 µg/L	2.2 µg/L	2.2 ng/mL	2.2 ng/mL
N-Acetyl-S-(N-methylcarbamoyl)-L-cysteine	99.6	2308	6.26 µg/L	6.26 µg/L	6.26 ng/mL	6.26 ng/mL
2-Aminothiazoline-4-carboxylic acid	93.6	2283	15 µg/L	15 µg/L	15 ng/mL	29.5 ng/mL
N-Acetyl-S-(benzyl)-L-cysteine	99.4	2314	0.5 µg/L	0.5 µg/L	0.5 ng/mL	0.5 ng/mL
N-Acetyl-S-(n-propyl)-L-cysteine	80.1	2279	1.2 µg/L	1.2 µg/L	1.2 ng/mL	1.2 ng/mL

N-Acetyl-S-(2-carboxyethyl)-L-cysteine	98.8	2305	6.96 µg/L	6.96 µg/L	6.96 ng/mL	6.96 ng/mL
N-Acetyl-S-(2-cyanoethyl)-L-cysteine	85.9	2298	0.5 µg/L	0.5 µg/L	0.5 ng/mL	0.5 ng/mL
N-Acetyl-S-(3,4-dihydroxybutyl)-L-cysteine	100	2254	5.25 µg/L	5.25 µg/L	5.25 ng/mL	5.25 ng/mL
N-Acetyl-S-(dimethylphenyl)-L-cysteine	0.4	1789	0.5 µg/L	0.5 µg/L	0.5 ng/mL	/
N-Acetyl-S-(2-carbamoyl-2-hydroxyethyl)-L-cysteine	43.3	2315	9.4 µg/L	9.4 µg/L	9.4 ng/mL	9.4 ng/mL
N-Acetyl-S-(2-hydroxyethyl)-L-cysteine	52.7	2308	0.791 µg/L	0.791 µg/L	0.791 ng/mL	0.791 ng/mL
N-Acetyl-S-(2-hydroxypropyl)-L-cysteine	94.9	2304	5.3 µg/L	5.3 µg/L	5.3 ng/mL	5.3 ng/mL
N-Acetyl-S-(3-hydroxypropyl)-L-cysteine	99.9	2275	13 µg/L	13 µg/L	13 ng/mL	13 ng/mL
Mandelic acid	98.5	2298	12 µg/L	12 µg/L	12 ng/mL	12 ng/mL
N-Acetyl-S-(1-hydroxymethyl)-2-propenyl)-L-cysteine	1.2	1806	0.7 µg/L	0.7 µg/L	0.7 ng/mL	/
N-Acetyl-S-(2-hydroxy-3-butenyl)-L-cysteine	15.2	1820	0.7 µg/L	0.7 µg/L	0.7 ng/mL	/
N-Acetyl-S-(4-hydroxy-2-butenyl)-L-cysteine	96	2286	0.6 µg/L	0.6 µg/L	0.6 ng/mL	0.6 ng/mL
N-Acetyl-S-(phenyl-2-hydroxyethyl)-L-cysteine	39.7	2282	0.7 µg/L	0.7 µg/L	0.7 ng/mL	1 ng/mL
Phenylglyoxylic acid	99.1	2302	12 µg/L	12 µg/L	12 ng/mL	12 ng/mL
N-Acetyl-S-(phenyl)-L-cysteine	44.9	1794	0.6 µg/L	0.6 µg/L	0.6 ng/mL	/
N-Acetyl-S-(3-hydroxypropyl-1-methyl)-L-cysteine	100	2295	1.13 µg/L	1.7 µg/L	1.7 ng/mL	1.7 ng/mL
N-Acetyl-S-(trichlorovinyl)-L-cysteine	0.1	1814	3 µg/L	3 µg/L	3 ng/mL	/
2-Thioxothiazolidine-4-carboxylic acid	37.3	1708	11.2 µg/L	11.2 µg/L	/	11.2 ng/mL

N-Acetyl-S-(1-cyano-2-hydroxyethyl)-L-cysteine	24.3	1102	/	/	2.6 ng/mL	2.6 ng/mL
N-Acetyl-S-(2-hydroxy-3-methyl-3-buten-1-yl)-L-cysteine & N-Acetyl-S-(2-hydroxy-2-methyl-3-buten-1-yl)-L-cysteine	83.2	607	/	/	1.43 ng/mL	/
N-Acetyl-S-(4-hydroxy-2-methyl-2-buten-1-yl)-L-cysteine	82.3	1102	/	/	1.2 ng/mL	1.2 ng/mL

/ indicates that there is no relevant pollutant data for that year or the data for that year was not used. The detection limit of Cesium is lost but does not affect the analysis. The detection limit of Inorganic mercury in urine is close to 70%, so it was also included in analysis.

Table S2. Water intake and concentrations of pollutants.

Plain water drank yesterday (mean ± SD)	1329.78 ± 1312.94 grams			
**Pollutants**	Number of participants	Concentration (mean ± SD)	Number of participants	Creatinine-adjusted concentration (mean ± SD)
**Metals in blood**				
Lead	4984	1.06 ± 1.37 µg/dL	/	/
Cadmium	4984	0.45 ± 0.56 µg/L	/	/
Manganese	4983	10.38 ± 3.96 µg/L	/	/
Total mercury	4984	1.36 ± 2.27 µg/L	/	/
Cobalt	1158	0.18 ± 0.14 µg/L	/	/
Methyl mercury	4979	1.19 ± 2.23 µg/L	/	/
**Per- and polyfluoroalkyl substances in serum**				
Perfluorodecanoic acid	2179	0.26 ± 0.38 ng/mL	/	/
Perfluorooctanoic acid	567	2.37 ± 2.44 ng/mL	/	/
Perfluorooctane sulfonic acid	567	8.04 ± 9.43 ng/mL	/	/
Perfluorohexane sulfonic acid	2179	1.58 ± 2.21 ng/mL	/	/
Perfluorononanoic acid	2179	0.75 ± 0.70 ng/mL	/	/
Linear-perfluorooctanoic acid	1053	1.58 ± 2.15 ng/mL	/	/
Linear-perfluorooctane sulfonic acid	1053	3.87 ± 4.39 ng/mL	/	/
Perfluoromethylheptane sulfonic acid isomers	1053	1.43 ± 1.27 ng/mL	/	/
**Volatile organic compounds in blood**				
M-/p-Xylene	3135	0.13 ± 0.72 ng/mL	/	/
Toluene	1676	0.20 ± 0.42 ng/mL	/	/

**Metals in urine**				
Total arsenic	2336	15.41 ± 34.36 µg/L	2334	14.55 ± 36.50 µg/g
Dimethylarsinic acid	2340	5.14 ± 6.11µg/L	2338	4.97 ± 5.65 µg/g
Inorganic mercury	2338	0.49 ± 2.11 µg/L	2336	0.46 ± 1.76 µg/g
Barium	2338	1.82 ± 2.68 µg/L	2336	1.78 ± 3.26 µg/g
Cadmium	2338	0.24 ± 0.32 µg/L	2336	0.21 ± 0.24 µg/g
Cobalt	2338	0.53 ± 0.55 µg/L	2336	0.50 ± 0.46 µg/g
Cesium	2338	4.95 ± 3.58 µg/L	2336	4.55 ± 2.71 µg/g
Molybdenum	2337	51.34 ± 54.91 µg/L	2335	43.51 ± 35.95 µg/g
Lead	2338	0.47 ± 1.10 µg/L	2336	0.43 ± 0.98 µg/g
Antimony	2338	0.08 ± 0.33 µg/L	2336	0.07 ± 0.14 µg/g
Strontium	1836	123.35 ± 108.75 µg/L	1834	117.44 ± 91.86 µg/g
Thallium	2338	0.21 ± 0.22 µg/L	2336	0.19 ± 0.14 µg/g
Tin	2337	1.03 ± 3.88 µg/L	2335	0.82 ± 2.26 µg/g
Tungsten	2333	0.14 ± 0.73 µg/L	2331	0.11 ± 0.32 µg/g
Uranium	1836	0.0096 ± 0.0222 µg/L	1834	0.011 ± 0.087 µg/g
Nickel	501	1.52 ± 2.10 µg/L	501	1.32 ± 1.36 µg/g
**Polycyclic aromatic hydrocarbon metabolites in urine**				
1-Hydroxynaphthalene	1803	20097.26 ± 332871.82 ng/L	1801	19196.66 ± 303024.51 ng/g
2-Hydroxynaphthalene	1817	10525.59 ± 17699.67 ng/L	1815	8608.57 ± 10271.84 ng/g
3-Hydroxyfluorene	1828	308.79 ± 635.72 ng/L	1826	250.71 ± 441.17 ng/g
2-Hydroxyfluorene	1830	560.98 ± 1084.36 ng/L	1828	475.41 ± 997.13 ng/g
3-Hydroxyphenanthrene	620	126.93 ± 162.50 ng/L	619	103.34 ± 125.09 ng/g
1-Hydroxyphenanthrene	1830	187.44 ± 314.27 ng/L	1828	167.81 ± 488.76 ng/g
2-Hydroxyphenanthrene	620	112.41 ± 134.17 ng/L	619	96.18 ± 108.20 ng/g

1-Hydroxypyrene	1827	237.24 ± 431.35 ng/L	1825	214.14 ± 393.70 ng/g
9-Hydroxyfluorene	620	546.55 ± 1058.68 ng/L	619	464.48 ± 858.69 ng/g
4-Hydroxyphenanthrene	619	38.27 ± 51.58 ng/L	618	35.37 ± 52.36 ng/g
**Phthalates and plasticizer metabolites in urine**				
Mono(carboxynonyl) phthalate	2306	4.35 ± 20.08 ng/mL	2304	3.59 ± 23.22 µg/g
Mono(carboxyoctyl) phthalate	2306	39.14 ± 98.73 ng/mL	2304	30.63 ± 68.67 µg/g
Mono-2-ethyl-5-carboxypentyl phthalate	2306	17.62 ± 44.50 ng/mL	2304	16.31 ± 87.22 µg/g
Mono-n-butyl phthalate	2306	16.80 ± 27.51 ng/mL	2304	12.57 ± 26.92 µg/g
Mono-(3-carboxypropyl) phthalate	2306	6.11 ± 40.55 ng/mL	2304	4.33 ± 21.88 µg/g
Mono-ethyl phthalate	2306	161.84 ± 689.75 ng/mL	2304	125.79 ± 446.83 µg/g
Mono-(2-ethyl-5-hydroxyhexyl) phthalate	2306	11.85 ± 32.15 ng/mL	2304	10.52 ± 46.85 µg/g
Mono-(2-ethyl-5-oxohexyl) phthalate	2306	7.33 ± 16.86 ng/mL	2304	6.44 ± 25.00 µg/g
Mono-benzyl phthalate	2306	10.24 ± 18.98 ng/mL	2304	7.19 ± 10.49 µg/g
Mono-isobutyl phthalate	2306	14.81 ± 27.40 ng/mL	2304	11.14 ± 23.81 µg/g
Mono-2-hydroxy-iso-butyl phthalate	1107	4.91 ± 9.27 ng/mL	1107	3.80 ± 9.61 µg/g
Mono-3-hydroxy-n-butyl phthalate	1107	1.4 ± 2.21 ng/mL	1107	1.10 ± 1.32 µg/g
Mono-2-ethyl-5-carboxypentylterephthalate	513	89.44 ± 218.53 ng/mL	513	71.89 ± 151.63 µg/g
Mono-2-ethyl-5-hydroxyhexylterephthalate	513	23.04 ± 55.05 ng/mL	513	18.59 ± 56.04 µg/g
Mono-oxoisononyl phthalate	513	3.58 ± 15.94 ng/mL	513	2.55 ± 7.32 µg/g
**Personal care and consumer product chemicals and metabolites in urine**				
Benzophenone-3	1794	340.58 ± 2747.69 ng/mL	1791	304.65 ± 1776.45 µg/g
Bisphenol A	1794	2.78 ± 6.66 ng/mL	1791	2.17 ± 5.31 µg/g
Triclosan	1794	82.18 ± 249.84 ng/mL	1791	71.32 ± 196.92 µg/g
Methyl paraben	1794	207.85 ± 553.31 ng/mL	1791	169.33 ± 353.48 µg/g
Propyl paraben	1794	47.62 ± 121.05 ng/mL	1791	42.77 ± 108.65 µg/g

2,5-dichlorophenol	1794	111.57 ± 827.17 µg/L	1791	76.11 ± 494.54 µg/g
2,4-dichlorophenol	1794	3.48 ± 18.84 µg/L	1791	2.55 ± 12.57 µg/g
Bisphenol S	1173	1.29 ± 3.69 µg/L	1171	1.31 ± 9.22 µg/g
**DEET and metabolites in Urine**				
3-(Ethlycarbamoyl) benzoic acid	1183	408.51 ± 11200.31 µg/L	1182	180.82 ± 4382.58 µg/g
**Glyphosate in urine**				
Glyphosate	1479	0.43 ± 0.45 ng/mL	1479	0.40 ± 0.42 µg/g
**Organophosphate insecticides and dialkyl phosphate metabolites in urine**				
Dimethylphosphate	1664	3.53 ± 7.07 ng/mL	1664	2.83 ± 4.58 µg/g
Diethylphosphate	1677	4.21 ± 10.20 ng/mL	1677	3.13 ± 6.13 µg/g
Dimethylthiophosphate	1672	3.08 ± 9.10 ng/mL	1672	2.60 ± 6.86 µg/g
**Pyrethroids, herbicides, and organophosphorus metabolites in urine**				
2,4-dicholorphenoxyacetic acid	1773	0.50 ± 1.68 µg/L	1772	0.46 ± 1.28 µg/g
3-phenoxybenzoic acid	1770	1.81 ± 6.27 µg/L	1769	1.56 ± 6.68 µg/g
Para-Nitrophenol	1750	1.06 ± 2.11 µg/L	1749	0.94 ± 2.33 µg/g
3,5,6-trichloropyridinol	1209	1.55 ± 1.88 µg/L	1208	1.30 ± 1.37 µg/g
**Perchlorate, nitrate and thiocyanate in urine**				
Perchlorate	2320	3.70 ± 5.18 ng/mL	2318	3.57 ± 5.21 µg/g
Nitrate	2321	58016.59 ± 48457.33 ng/mL	2319	54633.99 ± 48460.19 µg/g
Thiocyanate	2317	2533.90 ± 4049.62 ng/mL	2315	2355.36 ± 3516.79 µg/g
**Volatile organic compound metabolites in urine**				
2-Methylhippuric acid	2236	70.47 ± 126.21 ng/mL	2234	64.17 ± 119.84 µg/g
3-Methylhippuric acid & 4-Methylhippuric acid	2313	473.98 ± 1143.80 ng/mL	2311	404.97 ± 863.54 µg/g

N-Acetyl-S-(2-carbamoylethyl)-L-cysteine	2276	100.79 ± 146.59 ng/mL	2274	80.88 ± 88.78 µg/g
N-Acetyl-S-(N-methylcarbamoyl)-L-cysteine	2308	242.49 ± 312.27 ng/mL	2306	212.37 ± 240.92 µg/g
2-Aminothiazoline-4-carboxylic acid	2283	176.68 ± 199.51 ng/mL	2281	161.38 ± 148.98 µg/g
N-Acetyl-S-(benzyl)-L-cysteine	2314	13.47 ± 47.60 ng/mL	2312	11.05 ± 29.16 µg/g
N-Acetyl-S-(n-propyl)-L-cysteine	2279	14.12 ± 37.90 ng/mL	2277	13.87 ± 57.86 µg/g
N-Acetyl-S-(2-carboxyethyl)-L-cysteine	2305	154.03 ± 209.27 ng/mL	2303	128.24 ± 137.33 µg/g
N-Acetyl-S-(2-cyanoethyl)-L-cysteine	2298	55.82 ± 149.18 ng/mL	2296	44.99 ± 103.12 µg/g
N-Acetyl-S-(3,4-dihydroxybutyl)-L-cysteine	2254	368.18 ± 288.55 ng/mL	2252	309.83 ± 142.62 µg/g
N-Acetyl-S-(2-hydroxypropyl)-L-cysteine	2304	71.37 ± 222.99 ng/mL	2302	71.01 ± 249.60 µg/g
N-Acetyl-S-(3-hydroxypropyl)-L-cysteine	2275	548.39 ± 1021.14 ng/mL	2273	483.51 ± 711.32 µg/g
Mandelic acid	2298	211.09 ± 434.58 ng/mL	2296	176.47 ± 271.20 µg/g
N-Acetyl-S-(4-hydroxy-2-butenyl)-L-cysteine	2286	13.59 ± 24.64 ng/mL	2284	11.98 ± 20.69 µg/g
Phenylglyoxylic acid	2302	284.49 ± 615.20 ng/mL	2300	237.38 ± 358.33 µg/g
N-Acetyl-S-(3-hydroxypropyl-1-methyl)-L-cysteine	2295	519.05 ± 915.62 ng/mL	2293	460.20 ± 698.48 µg/g
N-Acetyl-S-(2-hydroxy-3-methyl-3-buten-1-yl)-L-cysteine & N-Acetyl-S-(2-hydroxy-2-methyl-3-buten-1-yl)-L-cysteine	607	5.84 ± 7.03 ng/mL	606	4.77 ± 4.06 µg/g
N-Acetyl-S-(4-hydroxy-2-methyl-2-buten-1-yl)-L-cysteine	1102	17.67 ± 47.13 ng/mL	1101	13.73 ± 29.86 µg/g

SD: Standard Deviation; Only concentrations of pollutants with detection rate of 70% or more were summarized; Since a small number of people do not have urine creatinine data, the number of participants decreases slightly after adjustment for creatinine. Blood pollutant concentrations do not need to be adjusted for creatinine, and the corresponding space is replaced by /.

Table S3. Associations between water intake and creatinine-unadjusted pollutants analyzed by four methods.

Pollutants	Number of participants	Method 1		Method 2		Method 3		Method 4			Result
		B (95% CI)	R^2	B (95% CI)	R^2	B (95% CI)	R^2	Average rank of low water drinking group	Average rank of high water drinking group	*P* value	
**Metals in blood**											
Lead	4984	-0.006 (-0.032, 0.021)	0.057	0.017 (-0.008, 0.042)	0.207	0.041 (0.015, 0.067)	0.165	1988.52	1987.46	0.977	Not
Cadmium	4984	-0.034 (-0.045, -0.023)	0.027	-0.076 (-0.103, -0.049)	0.068	-0.051 (-0.078, -0.023)	0.045	2087.69	1885.1	< 0.001	-
Manganese	4983	0.003 (-0.072, 0.079)	0.063	0.021 (-0.006, 0.048)	0.061	0.021 (-0.006, 0.048)	0.061	1973.47	2001.99	0.433	Not
Total mercury	4984	0.1 (0.056, 0.144)	0.039	0.096 (0.069, 0.124)	0.046	0.116 (0.089, 0.144)	0.027	1886.34	2092.94	< 0.001	+
Cobalt	1158	0.003 (-0.003, 0.009)	0.101	0.027 (-0.027, 0.08)	0.194	0.044 (-0.011, 0.099)	0.186	454.17	476.88	0.196	Not
Methyl mercury	4979	0.098 (0.055, 0.141)	0.038	0.097 (0.068, 0.125)	0.042	0.119 (0.09, 0.147)	0.024	1889.81	2086.36	< 0.001	+
**Per- and polyfluoroalkyl substances in serum**											
Perfluorodecanoic acid	2179	0.01 (-0.001, 0.022)	0.030	0.033 (-0.007, 0.073)	0.051	0.059 (0.019, 0.1)	0.022	869.44	876.57	0.764	Not
Perfluorooctanoic acid	567	0.118 (-0.021, 0.258)	0.044	0.039 (-0.037, 0.116)	0.140	0.056 (-0.02, 0.132)	0.141	227.59	231.48	0.753	Not
Perfluorooctane sulfonic acid	567	-0.166 (-0.699, 0.366)	0.064	-0.036 (-0.111, 0.039)	0.167	-0.03 (-0.105, 0.045)	0.165	241.2	217.38	0.054	Not
Perfluorohexane sulfonic acid	2179	0.026 (-0.037, 0.089)	0.074	0.031 (-0.006, 0.067)	0.284	0.033 (-0.003, 0.07)	0.282	866.98	879.04	0.617	Not
Perfluorononanoic acid	2179	-0.008 (-0.028, 0.012)	0.045	-0.004 (-0.045, 0.037)	0.070	0.013 (-0.029, 0.054)	0.062	885.07	860.89	0.315	Not
Linear-perfluorooctanoic acid	1053	0.083 (-0.008, 0.174)	0.038	0.098 (0.043, 0.153)	0.178	0.081 (0.025, 0.136)	0.166	398.93	424.99	0.116	Not
Linear-perfluorooctane sulfonic acid	1053	-0.035 (-0.217, 0.146)	0.092	-0.003 (-0.057, 0.052)	0.218	0.009 (-0.045, 0.064)	0.202	422.11	403.65	0.266	Not
Perfluoromethylheptane sulfonic acid isomers	1053	-0.019 (-0.067, 0.028)	0.249	0.013 (-0.038, 0.064)	0.336	0.01 (-0.042, 0.061)	0.333	418.32	407.14	0.500	Not

**Volatile organic compounds in blood**											
M-/p-Xylene	3135	-0.006 (-0.024, 0.012)	0.001	-0.108 (-0.144, -0.071)	0.028	-0.111 (-0.148, -0.074)	0.028	1321.63	1171.18	< 0.001	-
Toluene	1676	-0.031 (-0.045, -0.017)	0.025	-0.112 (-0.159, -0.064)	0.024	-0.112 (-0.16, -0.064)	0.024	711.72	618.58	< 0.001	-
**Metals in urine**											
Total arsenic	2336	-0.966 (-1.942, 0.01)	0.003	-0.027 (-0.068, 0.013)	0.006	-0.029 (-0.07, 0.011)	0.006	957.48	917.65	0.111	Not
Dimethylarsinic acid	2340	-0.157 (-0.33, 0.016)	0.005	-0.045 (-0.087, -0.003)	0.011	-0.049 (-0.091, -0.008)	0.009	973.69	904.58	0.006	-
Inorganic mercury	2338	-0.015 (-0.075, 0.045)	0.003	-0.059 (-0.103, -0.015)	0.011	-0.062 (-0.106, -0.017)	0.010	969.69	906.87	0.01	-
Barium	2338	-0.126 (-0.202, -0.051)	0.015	-0.11 (-0.15, -0.071)	0.029	-0.13 (-0.17, -0.09)	0.022	999.71	875.44	< 0.001	-
Cadmium	2338	-0.027 (-0.036, -0.018)	0.082	-0.146 (-0.184, -0.108)	0.107	-0.152 (-0.19, -0.114)	0.104	1023.83	850.19	< 0.001	-
Cobalt	2338	-0.038 (-0.053, -0.023)	0.062	-0.129 (-0.168, -0.09)	0.052	-0.143 (-0.183, -0.104)	0.047	1007.69	867.09	< 0.001	-
Cesium	2338	-0.293 (-0.393, -0.193)	0.033	-0.13 (-0.17, -0.091)	0.047	-0.144 (-0.184, -0.105)	0.029	1014.94	859.50	< 0.001	-
Molybdenum	2337	-2.178 (-3.727, -0.629)	0.018	-0.073 (-0.113, -0.034)	0.055	-0.1 (-0.14, -0.061)	0.035	985.90	888.93	< 0.001	-
Lead	2338	-0.024 (-0.055, 0.007)	0.004	-0.126 (-0.165, -0.087)	0.057	-0.134 (-0.173, -0.094)	0.059	1014.79	859.66	< 0.001	-
Antimony	2338	-0.008 (-0.017, 0.002)	0.003	-0.146 (-0.184, -0.107)	0.083	-0.18 (-0.219, -0.141)	0.054	1019.93	854.28	< 0.001	-
Strontium	1836	-7.427 (-10.896, -3.959)	0.016	-0.128 (-0.173, -0.083)	0.027	-0.13 (-0.175, -0.085)	0.026	800.96	670.68	< 0.001	-
Thallium	2338	-0.007 (-0.013, -0.001)	0.017	-0.073 (-0.113, -0.034)	0.047	-0.1 (-0.14, -0.06)	0.016	978.84	897.29	0.001	-
Tin	2337	-0.157 (-0.267, -0.048)	0.013	-0.136 (-0.176, -0.096)	0.053	-0.158 (-0.198, -0.117)	0.026	1011.28	862.39	< 0.001	-
Tungsten	2333	-0.021 (-0.042, -0.001)	0.005	-0.103 (-0.142, -0.064)	0.074	-0.132 (-0.172, -0.093)	0.050	996.85	875.57	< 0.001	-
Uranium	1836	-0.001 (-0.001, 0)	0.008	-0.086 (-0.132, -0.039)	0.033	-0.117 (-0.164, -0.069)	0.019	771.82	701.36	0.001	-
Nickel	501	-0.216 (-0.345, -0.087)	0.030	-0.162 (-0.248, -0.076)	0.052	-0.183 (-0.269, -0.097)	0.043	222.24	180.35	< 0.001	-
**Polycyclic aromatic hydrocarbon metabolites in urine**											
1-Hydroxynaphthalene	1803	-11096.876 (-21862.355, -331.396)	0.006	-0.167 (-0.212, -0.122)	0.045	-0.172 (-0.217, -0.127)	0.046	802.97	644.33	< 0.001	-
2-Hydroxynaphthalene	1817	-1491.433 (-2054.902, -927.963)	0.031	-0.155 (-0.199, -0.111)	0.061	-0.187 (-0.232, -0.142)	0.036	800.29	656.57	< 0.001	-
3-Hydroxyfluorene	1828	-76.342 (-96.428, -56.257)	0.037	-0.2 (-0.244, -0.156)	0.072	-0.212 (-0.256, -0.168)	0.072	829.15	633.93	< 0.001	-
2-Hydroxyfluorene	1830	-115.4 (-149.814, -80.986)	0.027	-0.207 (-0.251, -0.163)	0.076	-0.225 (-0.269, -0.181)	0.069	826.81	638.18	< 0.001	-

3-Hydroxyphenanthrene	620	-17.533 (-26.479, -8.586)	0.034	-0.186 (-0.263, -0.109)	0.059	-0.206 (-0.282, -0.13)	0.055	280.59	222.60	< 0.001	-
1-Hydroxyphenanthrene	1830	-23.736 (-33.771, -13.701)	0.015	-0.162 (-0.206, -0.117)	0.053	-0.188 (-0.233, -0.144)	0.042	809.70	656.23	< 0.001	-
2-Hydroxyphenanthrene	620	-16.345 (-23.589, -9.101)	0.071	-0.18 (-0.256, -0.105)	0.099	-0.22 (-0.296, -0.144)	0.063	280.62	222.57	< 0.001	-
1-Hydroxypyrene	1827	-33.446 (-47.249, -19.643)	0.014	-0.174 (-0.219, -0.128)	0.041	-0.192 (-0.238, -0.146)	0.038	807.70	655.35	< 0.001	-
9-Hydroxyfluorene	620	-86.809 (-144.61, -29.009)	0.050	-0.162 (-0.237, -0.087)	0.098	-0.2 (-0.277, -0.124)	0.055	279.87	223.34	< 0.001	-
4-Hydroxyphenanthrene	619	-4.584 (-7.407, -1.761)	0.048	-0.156 (-0.233, -0.079)	0.085	-0.199 (-0.277, -0.121)	0.05	275.01	227.22	< 0.001	-
**Phthalates and plasticizer metabolites in urine**											
Mono(carboxynonyl) phthalate	2306	-0.417 (-0.995, 0.162)	0.002	-0.111 (-0.152, -0.071)	0.063	-0.144 (-0.186, -0.103)	0.032	980.89	871.42	< 0.001	-
Mono(carboxyoctyl) phthalate	2306	-3.441 (-6.267, -0.616)	0.015	-0.087 (-0.128, -0.047)	0.037	-0.111 (-0.152, -0.071)	0.019	963.62	888.27	0.002	-
Mono-2-ethyl-5-carboxypentyl phthalate	2306	-1.096 (-2.376, 0.184)	0.005	-0.099 (-0.139, -0.059)	0.031	-0.122 (-0.163, -0.081)	0.016	979.04	873.22	< 0.001	-
Mono-n-butyl phthalate	2306	-1.344 (-2.134, -0.555)	0.009	-0.124 (-0.165, -0.084)	0.042	-0.146 (-0.187, -0.105)	0.023	987.46	865	< 0.001	-
Mono-(3-carboxypropyl) phthalate	2306	-1.615 (-2.781, -0.449)	0.005	-0.107 (-0.147, -0.066)	0.037	-0.132 (-0.173, -0.09)	0.022	974.88	877.28	< 0.001	-
Mono-ethyl phthalate	2306	-4.099 (-23.974, 15.777)	0.002	-0.045 (-0.085, -0.004)	0.016	-0.064 (-0.105, -0.023)	0.005	946.97	904.54	0.088	Not
Mono-(2-ethyl-5-hydroxyhexyl) phthalate	2306	-0.763 (-1.688, 0.162)	0.004	-0.097 (-0.137, -0.057)	0.033	-0.121 (-0.162, -0.08)	0.018	979.62	872.66	< 0.001	-
Mono-(2-ethyl-5-oxohexyl) phthalate	2306	-0.506 (-0.99, -0.021)	0.006	-0.104 (-0.144, -0.063)	0.036	-0.128 (-0.169, -0.087)	0.017	977.96	874.27	< 0.001	-
Mono-benzyl phthalate	2306	-1.128 (-1.668, -0.588)	0.026	-0.158 (-0.197, -0.119)	0.075	-0.178 (-0.218, -0.137)	0.042	1001.36	851.42	< 0.001	-
Mono-isobutyl phthalate	2306	-1.216 (-2.002, -0.429)	0.009	-0.117 (-0.157, -0.077)	0.038	-0.136 (-0.176, -0.095)	0.019	982.80	869.54	< 0.001	-
Mono-2-hydroxy-iso-butyl phthalate	1107	-0.577 (-0.967, -0.186)	0.009	-0.172 (-0.231, -0.113)	0.042	-0.19 (-0.25, -0.13)	0.040	477.84	395.39	< 0.001	-
Mono-3-hydroxy-n-butyl phthalate	1107	-0.11 (-0.203, -0.017)	0.012	-0.173 (-0.235, -0.111)	0.048	-0.197 (-0.26, -0.135)	0.048	471.16	401.27	< 0.001	-
Mono-2-ethyl-5-carboxypentylterephthalate	513	2.861 (-10.991, 16.713)	0.040	-0.084 (-0.174, 0.005)	0.043	-0.076 (-0.165, 0.013)	0.041	202.73	190.14	0.272	Not
Mono-2-ethyl-5-hydroxyhexylterephthalate	513	-0.056 (-3.537, 3.426)	0.044	-0.059 (-0.149, 0.031)	0.039	-0.062 (-0.151, 0.028)	0.027	201.61	191.11	0.36	Not
Mono-oxoisononyl phthalate	513	-0.449 (-1.471, 0.574)	0.017	-0.068 (-0.159, 0.023)	0.025	-0.087 (-0.178, 0.004)	0.014	202.53	190.32	0.285	Not
**Personal care and consumer product chemicals and metabolites in urine**											
Benzophenone-3	1794	19.764 (-68.954, 108.481)	0.001	0.069 (0.025, 0.114)	0.049	0.072 (0.027, 0.117)	0.049	699.35	761.91	0.005	+
Bisphenol A	1794	-0.288 (-0.503, -0.074)	0.010	-0.165 (-0.21, -0.119)	0.074	-0.196 (-0.242, -0.149)	0.049	792.5	667.99	< 0.001	-

Triclosan	1794	0.167 (-7.89, 8.224)	0.004	-0.035 (-0.083, 0.013)	0.003	-0.045 (-0.094, 0.003)	0.003	742.09	718.81	0.29	Not
Methyl paraben	1794	-19.226 (-36.974, -1.478)	0.014	-0.038 (-0.082, 0.006)	0.081	-0.035 (-0.08, 0.009)	0.081	741.04	719.88	0.338	Not
Propyl paraben	1794	-1.624 (-5.495, 2.248)	0.020	-0.042 (-0.085, 0.001)	0.124	-0.04 (-0.084, 0.004)	0.123	739.52	721.41	0.412	Not
2,5-dichlorophenol	1794	-2.966 (-29.677, 23.746)	0.001	-0.127 (-0.172, -0.082)	0.037	-0.141 (-0.187, -0.095)	0.022	788.75	671.77	< 0.001	-
2,4-dichlorophenol	1794	-0.063 (-0.671, 0.545)	0.002	-0.11 (-0.155, -0.065)	0.027	-0.127 (-0.173, -0.081)	0.017	781.75	678.83	< 0.001	-
Bisphenol S	1173	-0.117 (-0.263, 0.03)	0.004	-0.136 (-0.194, -0.078)	0.051	-0.166 (-0.226, -0.106)	0.035	508.60	448.40	0.001	-
**DEET and metabolites in Urine**											
3-(Ethlycarbamoyl) benzoic acid	1183	279.994 (-171.553, 731.542)	0.003	-0.024 (-0.08, 0.033)	0.014	-0.036 (-0.093, 0.021)	0.004	476.9	466.49	0.557	Not
**Glyphosate in urine**											
Glyphosate	1479	-0.022 (-0.038, -0.005)	0.009	-0.129 (-0.183, -0.075)	0.026	-0.146 (-0.201, -0.091)	0.020	617.22	544.05	< 0.001	-
**Organophosphate insecticides and dialkyl phosphate metabolites in urine**											
Dimethylphosphate	1664	-0.175 (-0.417, 0.066)	0.002	-0.042 (-0.09, 0.007)	0.002	-0.05 (-0.099, -0.002)	0.003	677.46	646.94	0.146	Not
Diethylphosphate	1677	-0.326 (-0.673, 0.021)	0.003	-0.087 (-0.135, -0.039)	0.012	-0.104 (-0.152, -0.056)	0.011	700.91	633.01	0.001	-
Dimethylthiophosphate	1672	-0.002 (-0.312, 0.308)	0	0.015 (-0.033, 0.063)	0.001	0.003 (-0.045, 0.052)	0.000	660.36	669.51	0.664	Not
**Pyrethroids, herbicides, and organophosphorus metabolites in urine**											
2,4-dicholorphenoxyacetic acid	1773	0.038 (-0.017, 0.093)	0.002	-0.028 (-0.076, 0.02)	0.015	-0.055 (-0.103, -0.007)	0.007	716.67	696.81	0.356	Not
3-phenoxybenzoic acid	1770	-0.111 (-0.315, 0.094)	0.005	-0.073 (-0.119, -0.027)	0.017	-0.086 (-0.132, -0.04)	0.008	737.46	673.41	0.003	-
Para-Nitrophenol	1750	0.004 (-0.065, 0.074)	0.001	-0.062 (-0.108, -0.016)	0.012	-0.082 (-0.129, -0.035)	0.01	720.65	673.33	0.028	-
3,5,6-trichloropyridinol	1209	-0.033 (-0.107, 0.041)	0.006	-0.092 (-0.146, -0.037)	0.034	-0.113 (-0.169, -0.058)	0.022	503.93	454.13	0.005	-
**Perchlorate, nitrate and thiocyanate in urine**											
Perchlorate	2320	-0.312 (-0.459, -0.165)	0.011	-0.084 (-0.124, -0.044)	0.025	-0.101 (-0.141, -0.061)	0.018	982.07	880.53	< 0.001	-
Nitrate	2321	-2010.991 (-3385.557, -636.425)	0.009	-0.095 (-0.134, -0.055)	0.026	-0.105 (-0.145, -0.065)	0.018	991.25	872	< 0.001	-
Thiocyanate	2317	-547.313 (-660.15, -434.476)	0.045	-0.199 (-0.238, -0.161)	0.084	-0.226 (-0.265, -0.187)	0.065	1037.42	821.6	< 0.001	-
**Volatile organic compound metabolites in urine**											

2-Methylhippuric acid	2236	-8.971 (-12.584, -5.357)	0.022	-0.187 (-0.227, -0.147)	0.058	-0.2 (-0.24, -0.16)	0.060	1007.38	791.3	< 0.001	-
3-Methylhippuric acid & 4-Methylhippuric acid	2313	-65.523 (-97.949, -33.098)	0.013	-0.19 (-0.229, -0.151)	0.057	-0.204 (-0.243, -0.164)	0.057	1037.24	815.87	< 0.001	-
N-Acetyl-S-(2-carbamoylethyl)-L-cysteine	2276	-13.085 (-17.218, -8.953)	0.036	-0.192 (-0.231, -0.153)	0.088	-0.214 (-0.253, -0.175)	0.076	1029.28	797.27	< 0.001	-
N-Acetyl-S-(N-methylcarbamoyl)-L-cysteine	2308	-40.454 (-49.155, -31.754)	0.048	-0.205 (-0.244, -0.166)	0.073	-0.224 (-0.263, -0.185)	0.068	1043.16	803.9	< 0.001	-
2-Aminothiazoline-4-carboxylic acid	2283	-11.154 (-16.724, -5.584)	0.056	-0.088 (-0.127, -0.049)	0.074	-0.1 (-0.139, -0.06)	0.062	964.44	869.44	< 0.001	-
N-Acetyl-S-(benzyl)-L-cysteine	2314	-1.957 (-3.311, -0.603)	0.006	-0.113 (-0.153, -0.074)	0.039	-0.13 (-0.17, -0.09)	0.018	993.06	863	< 0.001	-
N-Acetyl-S-(n-propyl)-L-cysteine	2279	-1.007 (-2.094, 0.08)	0.005	-0.019 (-0.059, 0.022)	0.007	-0.031 (-0.072, 0.01)	0.003	922.09	909.52	0.61	Not
N-Acetyl-S-(2-carboxyethyl)-L-cysteine	2305	-21.204 (-27.056, -15.352)	0.044	-0.2 (-0.238, -0.162)	0.113	-0.228 (-0.267, -0.19)	0.083	1036.04	809.08	< 0.001	-
N-Acetyl-S-(2-cyanoethyl)-L-cysteine	2298	-13.664 (-17.876, -9.451)	0.026	-0.213 (-0.252, -0.174)	0.075	-0.22 (-0.259, -0.181)	0.072	1043.14	799.1	< 0.001	-
N-Acetyl-S-(3,4-dihydroxybutyl)-L-cysteine	2254	-29.731 (-37.735, -21.727)	0.075	-0.185 (-0.224, -0.146)	0.108	-0.217 (-0.256, -0.177)	0.071	1007.33	802.94	< 0.001	-
N-Acetyl-S-(2-hydroxypropyl)-L-cysteine	2304	-2.271 (-8.628, 4.085)	0.004	-0.139 (-0.178, -0.099)	0.046	-0.151 (-0.191, -0.112)	0.039	1003.52	847.02	< 0.001	-
N-Acetyl-S-(3-hydroxypropyl)-L-cysteine	2275	-81.273 (-110.317, -52.228)	0.021	-0.193 (-0.232, -0.154)	0.074	-0.207 (-0.246, -0.168)	0.071	1025.96	797.41	< 0.001	-
Mandelic acid	2298	-23.745 (-36.095, -11.395)	0.015	-0.206 (-0.245, -0.167)	0.082	-0.224 (-0.263, -0.185)	0.069	1031.35	807.68	< 0.001	-
N-Acetyl-S-(4-hydroxy-2-butenyl)-L-cysteine	2286	-2.83 (-3.525, -2.135)	0.034	-0.214 (-0.253, -0.175)	0.077	-0.228 (-0.267, -0.189)	0.074	1037.67	794.91	< 0.001	-
Phenylglyoxylic acid	2302	-31.961 (-49.441, -14.481)	0.014	-0.177 (-0.216, -0.138)	0.073	-0.193 (-0.232, -0.154)	0.059	1024.40	817.90	< 0.001	-
N-Acetyl-S-(3-hydroxypropyl-1-methyl)-L-cysteine	2295	-88.98 (-114.825, -63.135)	0.027	-0.186 (-0.225, -0.147)	0.068	-0.202 (-0.242, -0.163)	0.062	1030.00	810.48	< 0.001	-
N-Acetyl-S-(2-hydroxy-3-methyl-3-buten-1-yl)-L-cysteine & N-Acetyl-S-(2-hydroxy-2-methyl-3-buten-1-yl)-L-cysteine	607	-1.02 (-1.403, -0.638)	0.077	-0.25 (-0.324, -0.176)	0.142	-0.252 (-0.328, -0.176)	0.123	279.17	203.92	< 0.001	-
N-Acetyl-S-(4-hydroxy-2-methyl-2-buten-1-yl)-L-cysteine	1102	-4.71 (-6.648, -2.773)	0.025	-0.214 (-0.271, -0.158)	0.075	-0.222 (-0.28, -0.165)	0.071	494.08	384.30	< 0.001	-

For methods 1, 2, 3, and 4, please refer to the description of statistical methods in the text.

B: Unstandardized regression coefficient; CI: Confidence interval;

Result represents whether the pollutant is associated with drinking water and the direction of the association from the analysis results of the four methods. For each pollutant, only three or four analysis results are significant and in the same direction, and then the amount of drinking water is considered to be

significantly related to the pollutant;

- Represents a stable negative correlation between drinking water and the level of a pollutant; + represents a stable positive correlation between drinking water and the level of a pollutant; Not means the result has no stable significance.

Table S4. Associations between water intake and creatinine-adjusted pollutants in urine analyzed by four methods.

Pollutants	Number of participants	Method 1		Method 2		Method 3		Method 4			Result
		B (95% CI)	R^2	B (95% CI)	R^2	B (95% CI)	R^2	Average rank of low water drinking group	Average rank of high water drinking group	*P* value	
**Metals in urine**											
Total arsenic	2334	0.26 (-0.768, 1.288)	0.021	0.102 (0.063, 0.141)	0.068	0.133 (0.093, 0.172)	0.035	881.52	994.97	< 0.001	+
Dimethylarsinic acid	2338	0.235 (0.079, 0.391)	0.061	0.113 (0.075, 0.151)	0.107	0.15 (0.111, 0.189)	0.064	873.33	1008.05	< 0.001	+
Inorganic mercury	2336	0.025 (-0.025, 0.075)	0.01	0.073 (0.035, 0.111)	0.082	0.095 (0.057, 0.134)	0.063	893.63	984.46	< 0.001	+
Barium	2336	-0.017 (-0.11, 0.075)	0.005	0.029 (-0.011, 0.068)	0.028	0.037 (-0.003, 0.077)	0.021	921.25	955.54	0.17	Not
Cadmium	2336	-0.008 (-0.014, -0.001)	0.159	-0.024 (-0.058, 0.01)	0.271	0.004 (-0.031, 0.039)	0.247	946.28	929.33	0.498	Not
Cobalt	2336	0.004 (-0.008, 0.016)	0.174	0.028 (-0.006, 0.063)	0.262	0.056 (0.021, 0.091)	0.235	910.87	966.40	0.026	Not
Cesium	2336	0.061 (-0.011, 0.134)	0.116	0.058 (0.021, 0.095)	0.150	0.088 (0.051, 0.126)	0.117	897.30	980.61	0.001	+
Molybdenum	2335	1.103 (0.09, 2.116)	0.021	0.081 (0.041, 0.121)	0.027	0.088 (0.048, 0.128)	0.015	888.93	988.30	< 0.001	+
Lead	2336	0.003 (-0.024, 0.031)	0.008	0.036 (-0.001, 0.073)	0.133	0.068 (0.029, 0.106)	0.064	925.60	950.98	0.31	Not
Antimony	2336	0.001 (-0.003, 0.005)	0.001	0.012 (-0.028, 0.052)	0.021	0.016 (-0.023, 0.056)	0.016	921.87	954.89	0.187	Not
Strontium	1834	1.837 (-1.021, 4.695)	0.065	0.026 (-0.017, 0.07)	0.080	0.063 (0.018, 0.108)	0.028	723.14	750.57	0.216	Not
Thallium	2336	0.01 (0.006, 0.014)	0.059	0.1 (0.062, 0.138)	0.084	0.121 (0.082, 0.159)	0.079	865.53	1013.87	< 0.001	+
Tin	2335	-0.06 (-0.124, 0.004)	0.011	-0.016 (-0.055, 0.022)	0.075	-0.01 (-0.049, 0.028)	0.074	937.64	937.35	0.991	Not
Tungsten	2331	0.001 (-0.008, 0.01)	0.003	0.003 (-0.037, 0.043)	0.012	0.005 (-0.035, 0.045)	0.011	927.97	945.40	0.485	Not
Uranium	1834	-0.002 (-0.004, 0.001)	0.001	0.07 (0.026, 0.114)	0.037	0.069 (0.025, 0.114)	0.030	698.82	776.18	< 0.001	+
Nickel	501	-0.105 (-0.186, -0.024)	0.080	-0.053 (-0.134, 0.029)	0.143	-0.039 (-0.121, 0.043)	0.128	210.28	192.54	0.126	Not

**Polycyclic aromatic hydrocarbon metabolites in urine**												
1-Hydroxynaphthalene	1801	-10205.696 (-20008.442, -402.949)	0.007	-0.09 (-0.135, -0.045)	0.037	-0.069 (-0.114, -0.023)	0.012	762.66	685.04	< 0.001	-
2-Hydroxynaphthalene	1815	-633.972 (-959.972, -307.972)	0.038	-0.091 (-0.135, -0.047)	0.059	-0.1 (-0.145, -0.056)	0.06	754.65	702.85	0.019	-
3-Hydroxyfluorene	1826	-48.861 (-62.791, -34.931)	0.04	-0.131 (-0.176, -0.086)	0.030	-0.117 (-0.162, -0.071)	0.014	786.56	676.67	< 0.001	-
2-Hydroxyfluorene	1828	-74.713 (-106.505, -42.922)	0.02	-0.121 (-0.166, -0.076)	0.023	-0.109 (-0.154, -0.064)	0.016	779.96	685.52	< 0.001	-
3-Hydroxyphenanthrene	619	-4.031 (-10.959, 2.897)	0.026	-0.045 (-0.122, 0.033)	0.036	-0.019 (-0.097, 0.058)	0.025	255.93	246.93	0.487	Not
1-Hydroxyphenanthrene	1828	-13.981 (-29.668, 1.706)	0.007	-0.066 (-0.111, -0.022)	0.060	-0.057 (-0.101, -0.012)	0.05	751.49	715.56	0.104	Not
2-Hydroxyphenanthrene	619	-2.351 (-8.345, 3.642)	0.026	-0.019 (-0.096, 0.058)	0.056	-0.023 (-0.099, 0.053)	0.055	251.37	251.63	0.984	Not
1-Hydroxypyrene	1825	-16.879 (-29.498, -4.261)	0.012	-0.062 (-0.106, -0.018)	0.064	-0.047 (-0.091, -0.002)	0.045	746.66	717.59	0.189	-
9-Hydroxyfluorene	619	-13.814 (-61.483, 33.854)	0.022	-0.043 (-0.12, 0.034)	0.062	-0.05 (-0.126, 0.026)	0.058	255.51	247.36	0.529	Not
4-Hydroxyphenanthrene	618	1.302 (-1.585, 4.189)	0.038	0.027 (-0.049, 0.104)	0.071	0.017 (-0.058, 0.093)	0.068	245.70	256.49	0.404	Not
**Phthalates and plasticizer metabolites in urine**												
Mono(carboxynonyl) phthalate	2304	-0.17 (-0.84, 0.499)	0.001	-0.001 (-0.041, 0.04)	0.006	-0.006 (-0.047, 0.035)	0.003	919.21	929.66	0.674	Not
Mono(carboxyoctyl) phthalate	2304	-0.605 (-2.582, 1.372)	0.004	0.009 (-0.032, 0.049)	0.009	0.00006938 (-0.041, 0.041)	0.007	915.38	933.41	0.468	Not
Mono-2-ethyl-5-carboxypentyl phthalate	2304	1.174 (-1.342, 3.689)	0.001	0.044 (0.004, 0.084)	0.031	0.046 (0.006, 0.087)	0.03	909.78	938.87	0.241	Not
Mono-n-butyl phthalate	2304	-0.148 (-0.92, 0.624)	0.012	-0.026 (-0.065, 0.014)	0.058	-0.025 (-0.065, 0.015)	0.058	939.36	909.99	0.237	Not
Mono-(3-carboxypropyl) phthalate	2304	-0.64 (-1.271, -0.009)	0.002	-0.003 (-0.044, 0.037)	0.012	-0.008 (-0.049, 0.033)	0.012	921.76	927.18	0.827	Not
Mono-ethyl phthalate	2304	7.118 (-5.75, 19.986)	0.004	0.042 (0.002, 0.082)	0.028	0.044 (0.004, 0.085)	0.028	899.67	948.75	0.048	+
Mono-(2-ethyl-5-hydroxyhexyl) phthalate	2304	0.145 (-1.207, 1.496)	0.000	0.018 (-0.022, 0.058)	0.019	0.017 (-0.023, 0.058)	0.018	915.59	933.20	0.478	Not
Mono-(2-ethyl-5-oxohexyl) phthalate	2304	0.132 (-0.589, 0.853)	0.001	0.023 (-0.017, 0.062)	0.040	0.022 (-0.018, 0.062)	0.040	913.87	934.88	0.398	Not
Mono-benzyl phthalate	2304	-0.392 (-0.691, -0.094)	0.027	-0.069 (-0.108, -0.029)	0.039	-0.072 (-0.112, -0.032)	0.032	957.79	892.00	0.008	-
Mono-isobutyl phthalate	2304	-0.349 (-1.035, 0.337)	0.002	0.005 (-0.035, 0.045)	0.035	0.009 (-0.031, 0.05)	0.035	920.91	928.01	0.775	Not

Mono-2-hydroxy-iso-butyl phthalate	1107	-0.281 (-0.687, 0.125)	0.005	-0.059 (-0.116, -0.001)	0.074	-0.047 (-0.105, 0.011)	0.068	449.00	420.79	0.098	Not
Mono-3-hydroxy-n-butyl phthalate	1107	0.016 (-0.038, 0.07)	0.056	-0.015 (-0.072, 0.041)	0.104	-0.009 (-0.066, 0.049)	0.089	439.35	429.29	0.555	Not
Mono-2-ethyl-5-carboxypentylterephthalate	513	0.81 (-8.928, 10.548)	0.014	0.046 (-0.044, 0.136)	0.023	0.075 (-0.015, 0.165)	0.023	191.24	200.14	0.437	Not
Mono-2-ethyl-5-hydroxyhexylterephthalate	513	0.846 (-2.769, 4.461)	0.005	0.049 (-0.042, 0.14)	0.017	0.062 (-0.029, 0.152)	0.014	189.78	201.41	0.310	Not
Mono-oxoisononyl phthalate	513	0.068 (-0.402, 0.537)	0.018	0.088 (-0.001, 0.177)	0.038	0.085 (-0.004, 0.174)	0.037	184.93	205.64	0.071	Not
**Personal care and consumer product chemicals and metabolites in urine**											
Benzophenone-3	1791	46.907 (-10.204, 104.019)	0.012	0.138 (0.095, 0.181)	0.114	0.151 (0.107, 0.195)	0.106	670.39	788.01	< 0.001	+
Bisphenol A	1791	-0.016 (-0.188, 0.155)	0.003	-0.072 (-0.117, -0.027)	0.021	-0.07 (-0.116, -0.024)	0.021	753.16	704.68	0.028	-
Triclosan	1791	4.38 (-1.925, 10.684)	0.02	0.045 (0, 0.09)	0.023	0.052 (0.006, 0.098)	0.015	709.61	748.52	0.078	Not
Methyl paraben	1791	4.369 (-6.769, 15.508)	0.050	0.031 (-0.011, 0.073)	0.158	0.046 (0.004, 0.089)	0.148	712.03	746.09	0.122	Not
Propyl paraben	1791	1.789 (-1.641, 5.218)	0.047	0.021 (-0.02, 0.062)	0.186	0.034 (-0.008, 0.076)	0.178	714.26	743.84	0.18	Not
2,5-dichlorophenol	1791	2.482 (-13.494, 18.458)	0.002	-0.069 (-0.115, -0.024)	0.012	-0.071 (-0.117, -0.025)	0.010	762.61	695.16	0.002	-
2,4-dichlorophenol	1791	0.074 (-0.332, 0.479)	0.003	-0.004 (-0.049, 0.041)	0.022	-0.002 (-0.047, 0.044)	0.020	737.92	720.02	0.417	Not
Bisphenol S	1171	-0.203 (-0.569, 0.164)	0.004	-0.044 (-0.1, 0.011)	0.011	-0.055 (-0.112, 0.002)	0.011	486.82	468.22	0.297	Not
**DEET and metabolites in Urine**											
3-(Ethlycarbamoyl) benzoic acid	1182	104.711 (-72.161, 281.583)	0.003	0.03 (-0.027, 0.087)	0.002	0.045 (-0.012, 0.102)	0.003	461.91	482.3	0.251	Not
**Glyphosate in urine**											
Glyphosate	1479	0.022 (0.007, 0.037)	0.043	0.046 (-0.004, 0.096)	0.041	0.069 (0.019, 0.12)	0.035	562.43	595.55	0.092	Not
**Organophosphate insecticides and dialkyl phosphate metabolites in urine**											
Dimethylphosphate	1664	0.13 (-0.025, 0.285)	0.021	0.056 (0.009, 0.104)	0.045	0.068 (0.02, 0.116)	0.025	645.93	677.67	0.131	Not
Diethylphosphate	1677	-0.006 (-0.213, 0.202)	0.011	0.028 (-0.02, 0.075)	0.029	0.037 (-0.011, 0.084)	0.023	666.06	666.93	0.967	Not
Dimethylthiophosphate	1672	0.272 (0.039, 0.504)	0.010	0.087 (0.04, 0.135)	0.037	0.096 (0.048, 0.144)	0.025	633.87	695.26	0.004	+
**Pyrethroids, herbicides, and organophosphorus metabolites in urine**											

2,4-dicholorphenoxyacetic acid	1772	0.067 (0.025, 0.108)	0.009	0.096 (0.051, 0.141)	0.036	0.116 (0.07, 0.162)	0.030	668.66	746.43	< 0.001	+
3-phenoxybenzoic acid	1769	0.01 (-0.208, 0.228)	0.007	0.005 (-0.041, 0.051)	0.025	0.022 (-0.024, 0.068)	0.025	709.08	701.78	0.737	Not
Para-Nitrophenol	1749	0.046 (-0.03, 0.123)	0.012	0.058 (0.012, 0.104)	0.034	0.074 (0.027, 0.12)	0.020	679.85	714.93	0.104	Not
3,5,6-trichloropyridinol	1208	0.056 (0.002, 0.109)	0.022	0.063 (0.008, 0.118)	0.039	0.075 (0.019, 0.13)	0.03	465.63	491.37	0.149	+
**Perchlorate, nitrate and thiocyanate in urine**											
Perchlorate	2318	-0.051 (-0.198, 0.096)	0.018	0.054 (0.014, 0.093)	0.054	0.069 (0.03, 0.109)	0.029	898.07	966.55	0.006	+
Nitrate	2319	2238.924 (900.888, 3576.96)	0.062	0.077 (0.039, 0.115)	0.108	0.102 (0.063, 0.141)	0.068	881.99	984.37	< 0.001	+
Thiocyanate	2315	-347.471 (-446.607, -248.334)	0.023	-0.107 (-0.147, -0.067)	0.015	-0.102 (-0.143, -0.062)	0.014	976.26	883.6	< 0.001	-
**Volatile organic compound metabolites in urine**											
2-Methylhippuric acid	2234	-2.425 (-5.879, 1.028)	0.011	-0.105 (-0.145, -0.065)	0.026	-0.099 (-0.14, -0.058)	0.014	957.21	841.48	< 0.001	-
3-Methylhippuric acid & 4-Methylhippuric acid	2311	-21.198 (-45.72, 3.323)	0.011	-0.125 (-0.164, -0.085)	0.027	-0.118 (-0.158, -0.078)	0.016	993.57	859.46	< 0.001	-
N-Acetyl-S-(2-carbamoylethyl)-L-cysteine	2274	-7.089 (-9.612, -4.566)	0.022	-0.11 (-0.15, -0.07)	0.017	-0.109 (-0.149, -0.069)	0.013	977.78	849.19	< 0.001	-
N-Acetyl-S-(N-methylcarbamoyl)-L-cysteine	2306	-23.12 (-29.776, -16.465)	0.065	-0.121 (-0.16, -0.081)	0.061	-0.113 (-0.152, -0.074)	0.054	984.67	863.32	< 0.001	-
2-Aminothiazoline-4-carboxylic acid	2281	1.736 (-2.214, 5.686)	0.150	0.017 (-0.019, 0.052)	0.24	0.03 (-0.005, 0.066)	0.236	891.18	944.01	0.033	Not
N-Acetyl-S-(benzyl)-L-cysteine	2312	-0.579 (-1.408, 0.249)	0.008	0.002 (-0.037, 0.041)	0.054	0.015 (-0.024, 0.054)	0.052	922.44	934.84	0.619	Not
N-Acetyl-S-(n-propyl)-L-cysteine	2277	-0.217 (-1.878, 1.444)	0.004	0.079 (0.039, 0.119)	0.026	0.087 (0.047, 0.127)	0.017	866.48	966.64	< 0.001	+
N-Acetyl-S-(2-carboxyethyl)-L-cysteine	2303	-9.275 (-13.155, -5.396)	0.025	-0.099 (-0.139, -0.059)	0.021	-0.107 (-0.147, -0.067)	0.021	977.14	869.05	< 0.001	-
N-Acetyl-S-(2-cyanoethyl)-L-cysteine	2296	-10.645 (-13.543, -7.746)	0.036	-0.128 (-0.168, -0.089)	0.025	-0.11 (-0.15, -0.07)	0.013	992.35	849.86	< 0.001	-
N-Acetyl-S-(3,4-dihydroxybutyl)-L-cysteine	2252	-5.481 (-9.521, -1.441)	0.037	-0.065 (-0.105, -0.025)	0.042	-0.065 (-0.105, -0.025)	0.042	929.24	882.68	0.058	-
N-Acetyl-S-(2-hydroxypropyl)-L-cysteine	2302	3.385 (-3.743, 10.514)	0.001	-0.028 (-0.068, 0.012)	0.017	-0.008 (-0.048, 0.033)	0.004	937.60	913.88	0.34	Not
N-Acetyl-S-(3-hydroxypropyl)-L-cysteine	2273	-45.995 (-66.125, -25.865)	0.032	-0.083 (-0.123, -0.043)	0.025	-0.068 (-0.109, -0.028)	0.009	959.06	865.03	< 0.001	-
Mandelic acid	2296	-7.321 (-15.067, 0.424)	0.007	-0.102 (-0.142, -0.063)	0.054	-0.081 (-0.121, -0.042)	0.037	966.76	873.16	< 0.001	-
N-Acetyl-S-(4-hydroxy-2-butenyl)-L-cysteine	2284	-1.845 (-2.429, -1.261)	0.032	-0.123 (-0.163, -0.083)	0.027	-0.111 (-0.151, -0.071)	0.02	979.16	854.05	< 0.001	-

Phenylglyoxylic acid	2300	-8.248 (-18.488, 1.993)	0.004	-0.069 (-0.108, -0.03)	0.059	-0.041 (-0.081, -0.001)	0.036	947.92	895.90	0.036	-
N-Acetyl-S-(3-hydroxypropyl-1-methyl)-L-cysteine	2293	-51.248 (-70.903, -31.593)	0.034	-0.074 (-0.114, -0.034)	0.026	-0.054 (-0.094, -0.014)	0.013	958.06	883.40	0.003	-
N-Acetyl-S-(2-hydroxy-3-methyl-3-buten-1-yl)-L-cysteine & N-Acetyl-S-(2-hydroxy-2-methyl-3-buten-1-yl)-L-cysteine	606	-0.343 (-0.567, -0.119)	0.053	-0.151 (-0.23, -0.073)	0.042	-0.142 (-0.222, -0.062)	0.032	261.94	219.76	0.001	-
N-Acetyl-S-(4-hydroxy-2-methyl-2-buten-1-yl)-L-cysteine	1101	-2.855 (-4.079, -1.631)	0.031	-0.104 (-0.162, -0.046)	0.022	-0.093 (-0.152, -0.034)	0.014	464.09	413.14	0.003	-

Pollutant concentrations were adjusted for creatinine and then analyzed using the same four methods as in Table S3;
For methods 1, 2, 3, and 4, please refer to the description of statistical methods in the text;
B: Unstandardized regression coefficient; CI: Confidence interval;
Result represents whether the pollutant is associated with drinking water and the direction of the association from the analysis results of the four methods. For each pollutant, only three or four analysis results are significant and in the same direction, and then the amount of drinking water is considered to be significantly related to the pollutant;
- Represents a stable negative correlation between drinking water and the level of a pollutant; + represents a stable positive correlation between drinking water and the level of a pollutant; Not means the result has no stable significance.
